# p53 Proteoforms and Intrinsic Disorder: An Illustration of the Protein Structure–Function Continuum Concept

**DOI:** 10.3390/ijms17111874

**Published:** 2016-11-10

**Authors:** Vladimir N. Uversky

**Affiliations:** 1 Department of Molecular Medicine and USF Health Byrd Alzheimer’s Research Institute, Morsani College of Medicine, University of South Florida, 12901 Bruce B. Downs Blvd. MDC07, Tampa, FL 33612, USA; vuversky@health.usf.edu; Tel.: +1-813-974-5816; Fax: +1-813-974-7357; 2 Laboratory of Structural Dynamics, Stability and Folding of Proteins, Institute of Cytology, Russian Academy of Sciences, 4 Tikhoretsky Ave., 194064 St. Petersburg, Russia

**Keywords:** p53, proteoform, protein structure–function continuum, cancer, mutation, posttranslational modification, intrinsically disordered protein, protein–protein interaction, alternative splicing

## Abstract

Although it is one of the most studied proteins, p53 continues to be an enigma. This protein has numerous biological functions, possesses intrinsically disordered regions crucial for its functionality, can form both homo-tetramers and isoform-based hetero-tetramers, and is able to interact with many binding partners. It contains numerous posttranslational modifications, has several isoforms generated by alternative splicing, alternative promoter usage or alternative initiation of translation, and is commonly mutated in different cancers. Therefore, p53 serves as an important illustration of the protein structure–function continuum concept, where the generation of multiple proteoforms by various mechanisms defines the ability of this protein to have a multitude of structurally and functionally different states. Considering p53 in the light of a proteoform-based structure–function continuum represents a non-canonical and conceptually new contemplation of structure, regulation, and functionality of this important protein.

## 1. Introduction

If one were to look for the most representative protein that could successfully challenge the more than century-old structure–function paradigm represented by the famous lock-and-key model, the search would not be too long. In fact, even a superficial glance through the currently available information related to the most studied human protein, tumor protein p53 (which is the subject of almost 83,000 papers), would indicate that no further search is needed, since the appropriate candidate is found. Actually, with its countless biological functions and well-known capability to interact with a myriad of unrelated binding partners, p53 acts as a polyfunctional multibinder, whose functional molecular mechanisms are clearly opposed to the lock-and-key-like functionality described for many globular proteins. This rich functional spectrum of p53 is a reflection of the richness and complexity of its structure, with multiple proteoforms generated due to the presence of intrinsically disordered regions, numerous posttranslational modifications, and multiple isoforms created by alternative splicing, alternative promoter usage, or alternative initiation of translation, and the ability of p53 to homo-tetramerize. All these factors play a role in defining the biological multifarious nature of this protein. Therefore, for understanding its multifunctionality and roles in carcinogenesis, p53 should be considered through the prism of the proteoform-based protein structure–function continuum, and not in terms of the classical lock-and-key model. This article uses several novel concepts, such as protein intrinsic disorder, spatiotemporal structural heterogeneity, proteoforms, and the structure–function continuum to shed some light on p53 and its enigmatic, multifaceted functionality.

## 2. Intrinsically Disordered Proteins and Intrinsically Disordered Protein Regions

For over a century, the lock-and-key model of interaction between protein and substrate or between two proteins [1] has dominated much of molecular biology’s history, creating the basis for modern protein science [2,3]. However, as verified by an increasing number of experimental observations, more and more proteins or their regions have been found to lack a unique 3D structure in their native states under physiological conditions. These proteins and regions, known respectively as intrinsically disordered proteins (IDPs) and intrinsically disordered protein regions (IDPRs) among different other names [4,5,6,7,8,9,10], exist in their functional states as conformational ensembles containing a large number of widely different conformations that are in rapid interconversion on different time scales. During the past 15 years or so, the protein intrinsic disorder phenomenon went from being at first rejected and ignored to becoming well-accepted by the scientific community. This is because IDPs/IDPRs have broken the major rule of protein science and structural biology, the protein structure–function paradigm. In fact, unlike ordered proteins, IDPs/IDPRs fail to form unique 3D structures in their functional states and exist as highly dynamic structural ensembles, either at the secondary or the tertiary level [4,6,7,11,12,13,14,15]. Furthermore, disorder bestows a number of properties on IDPs/IDPRs that would be difficult or even impossible for the folded proteins to have. Computational analyses revealed that the putative fraction of sequences with predicted long disordered segments (30 or longer) increases in the order: Bacteria ≈ Archeae << Eukaryota [8,16,17,18,19]. The increased amount of disorder in eukaryotes may be related to the increase in their cellular signaling [4]. Disordered proteins possess multiple specific features and can be engaged in novel types of regulation, making them appropriate candidates for signaling functions [4,7,12,20,21,22,23]. 

Every member of a given population of disordered protein molecules assumes distinct conformations that vary significantly over time. Some disordered regions are positively charged, while others are negatively charged. Some disordered regions contain many different amino acids (i.e., they have high sequence complexity) while other disordered regions have just a few different amino acids (i.e., they have low sequence complexity). Such proteins and regions have two categories as their extremes: extended and collapsed IDPs/IDPRs, with extended IDPs having high solvent exposure and collapsed IDPs having a restricted range of motion relative to extended disorder [2,3,6,14,24,25]. The native molten globule can serve as an example of collapsed disorder [24]. The heterogeneous spatiotemporal structure of IDPs/IDPRs can be described as a set of foldons, inducible foldons, semi-foldons, non-foldons, and unfoldons [14]. 

Although they are without stable 3D structures, IDPs/IDPRs play a number of crucial functional roles in living organisms, especially in vital biological processes, such as control, signaling, recognition, and regulation [2,3,7,20,21,25,26]. The functions of IDPs/IDPRs are believed to complement the biological activities of ordered proteins and domains. In fact, according to the statistical analysis of the SwissProt database, about 240 of 710 SwissProt functional keywords were shown to be strongly positively correlated with intrinsic disorder, whereas 300 functional keywords mostly characterizing various catalytic activities were strongly negatively correlated with intrinsic disorder [27]. The aforementioned structural heterogeneity of IDPs/IDPRs defines their ability to be promiscuous binders [20,21,28,29,30]. This property explains why intrinsic disorder is a common feature of hubs in protein–protein interaction networks [31] and why it is frequently found in signaling proteins [32]. Furthermore, many IDPs and IDPRs are known to be involved in the pathogenesis of various human diseases, such as cancer, cardiovascular diseases, amyloidoses, several neurodegenerative diseases, diabetes, etc. [33,34].

## 3. Proteoforms

**Figure 1:**
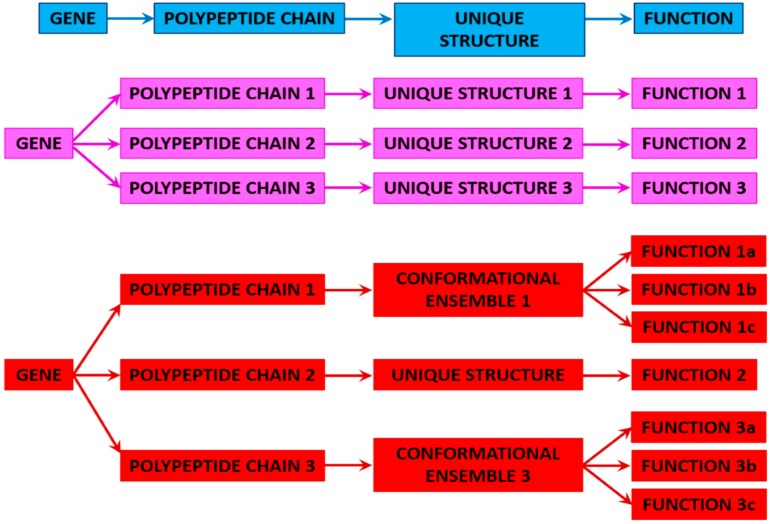
Illustration of the increase in complexity of the gene–protein relationship, starting from an oversimplified classic “one-gene–one-protein–one-function” model (**top** part, blue) and moving to more complex “one-gene–many-proteins–many-functions” scenarios caused by alternative splicing and PTMs affecting ordered proteins (**middle** part, pink) or intrinsically disordered and hybrid proteins containing ordered and intrinsically disordered domains (**bottom** part, red). Reproduced with permission from [45].

It is recognized now that the influential “one-gene–one-enzyme” hypothesis, according to which each gene is responsible for producing a single enzyme that in turn affects a single step in a metabolic pathway [35], is an oversimplification. In fact, the need for the replacement of this paradigm by new concepts was reinforced by the accumulation of numerous pieces of evidence “which do not fit into the widely accepted theoretical framework of how Nature functions” [36]. Probably one of the most compelling illustrations of the need to reconsider the “one-gene–one-protein–one-function” model is given by the contradiction between the number of protein-coding genes in a human cell, which is approaching (2.0–2.5) × 10^4^ [37], and the total number of human proteins with different functions, which is mounting to >2 × 10^6^ [38,39,40,41,42]. There are several means by which this increase in protein diversity leading to the “one-gene–many-proteins” or “one-gene–many-functions” concept (see Figure 1) can be reached, ranging from alternative splicing, to allelic variations, to other gene-affecting pre-translational mechanisms, and to a variety of post-translational modifications (PTMs) [38,39,40,41,42]. Therefore, it seems that the major part of the complexity of the biological machinery is determined by protein variation rather than results from a high number of distinct genes [43]. In line with this hypothesis, it was pointed out that variations within populations, cell and tissue types, and subcellular localization can arise from the differences among highly related, but chemically different, protein molecules [44], proteoforms (see below). Here, allelic variations (i.e., single or multiple point mutations, indels, SNPs), alternative splicing of mRNA, and numerous PTMs define complexity on the DNA, mRNA, and protein levels, respectively [44]. This multilevel complexity drives the creation of different protein molecules with diverse biological functions and the ability to affect various biological processes that range from gene regulation and cell signaling to the regulation of various pathways and protein complexes [44].

To account for the fact that all this variability is derived from a single gene, the term “*proteoform*” was proposed to “designate all of the different molecular forms in which the protein product of a single gene can be found, including changes due to genetic variations, alternatively spliced mRNA and PTMs” [44].

### 3.1. Ordered and Intrinsically Disordered Proteoforms

Since many PTM sites are known to be preferentially found within the regions of intrinsic disorder [46,47], since mRNA regions affected by alternative splicing predominantly encodes IDPRs [48], and since IDPs/IDPRs are known to be highly promiscuous binders [4,6,7,12,13,14,15,20,21,22,23,24,28,29,49,50,51,52,53,54,55], disordered proteins represent a very rich source of proteoforms. In fact, one might find numerous examples of new “one gene–many proteins–many functions” paradigm predominantly among IDPs. 

One should keep in mind, though, that the presence of intrinsic disorder adds a further level of complexity to the proteoform concept. In fact, the aforementioned definition of proteoform as a collection of different molecular forms in which the protein product of a single gene can be found, including changes due to genetic variations, alternatively spliced mRNA, and PTMs [44] is based on the “rigid” view of a protein molecule as a biological entity with a unique structure that can be modified by mutations, alternative splicing, and PTMs. 

This is because of the long-standing and broadly accepted structure–function paradigm, according to which the specific functionality of a given protein is determined by its unique 3D structure, where the protein and substrate have to fit to each other like a lock and key in order to exert a chemical effect on each other. In reality, not all proteins are structured throughout their entire lengths and many proteins are, in fact, highly flexible or structurally disordered as a whole or contain substantial IDPRs (see above). Furthermore, even though well-folded proteins are characterized by unique 3D structures, these proteins cannot be considered as completely rigid, rock-like entities. On the contrary, the importance of conformational flexibility and the need of structural dynamics for the successful functionality of globular proteins (even enzymes) have been emphasized in many studies over the past 55 years (e.g., [56,57,58,59,60,61,62,63,64,65,66,67,68]). 

**Figure 2:**
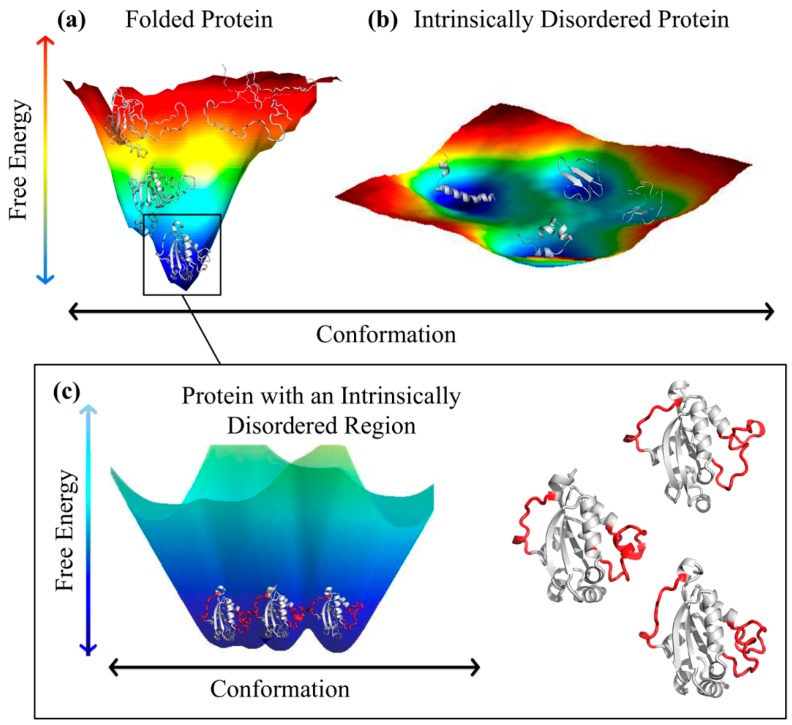
Schematic representation of the energy landscapes for (**a**) an ordered protein; (**b**) an IDP; and (**c**) a close-up view of the bottom of the funnel-like energy landscape of a hybrid protein containing ordered domains (shown in white) and IDPRs (shown in red). Reproduced from [76], which is an open access article distributed under the Creative Commons Attribution License that permits unrestricted use, distribution, and reproduction in any medium, provided that the original work is properly cited.

As a matter of fact, the biological functions of ordered proteins are known to be heavily dependent on the internal dynamics of enzymes, where individual amino acid residues, groups of amino acids, and even entire domains move relative to each other in a wide range of time scales, from femtoseconds to seconds, to facilitate catalytic activity [58,64,65]. It was also emphasized that functional conformational changes and the allosteric behavior of globular proteins can rely on the existence of conformational substates, which can be described as the atomic displacements leading to the formation and interconversion of different local configurations of the same overall protein structure [69,70,71,72,73,74,75]. This idea is illustrated by Figure 2, which represents the potential energy landscapes of ordered and disordered proteins [76]. The energy landscape of ordered proteins is characterized by a specific funnel-like shape, where a broad mouth at the top represents a set of unfolded conformations and the narrow end at the bottom shows the lowest energy state that corresponds to the native structure [77,78,79,80,81]. On the contrary, IDPs are characterized by a relative flat but rough energy landscape with multiple local energy minima separated by small barriers [14,33,76,82]. Finally, careful analysis of the bottom of the funnel-shaped energy landscape revealed that, for many proteins, the surface of the energy minimum is actually not smooth, being rough because of the presence of many smaller minima corresponding to different states sampled by the protein (see Figure 2). This is definitely the case for so-called hybrid proteins containing ordered domains and IDPRs and even for “normal” ordered proteins whose structures have been solved via X-ray crystallography or by nuclear magnetic resonance (NMR) and which are considered to be folded, but still often contain both ordered regions and intrinsically disordered regions lacking a stable tertiary structure [76,83].

### 3.2. Different Types of Proteoforms: A General Overview

Therefore, even without mutations, PTMs, or alternative splicing, any given protein can be considered as a basic (or intrinsic, or conformational) proteoform because it does exist as a dynamic conformational ensemble, members of which have different structures (their structural differences could be rather subtle, as in the case of ordered proteins, or rather substantial, as in the case of IDPs/IDPRs) and potentially can have different functions. Such a conformational proteoform is different from the inducible proteoform originating due to the various alterations (mutations, PTMs, or consequences of alternative splicing) of the canonical protein sequence and representing a mixture of these various forms. Obviously, any member of the inducible (or modified) proteoform (i.e., any mutated, modified, or alternatively spliced form) is a conformational proteoform itself since it also represents a structural ensemble. Finally, since protein function, interaction with specific partners, or placement inside the natural cellular environment (which is extremely crowded, characterized by the presence of high concentrations of various biological macromolecules [84,85,86], has limited available volume [87], and restricted amounts of free water [84,88,89,90,91,92]) can affect the structural ensemble of both basic and induced proteoforms, functionality per se can be considered as a factor generating new functioning proteoforms. 

Looking at all these considerations constituting the foundation for the “one gene–many proteins–many functions” paradigm, one can clearly see that the old protein structure–function model, according to which a unique function can only be conducted by a protein with a unique 3D structure, information about which is encoded in a unique amino acid sequence, should be substituted with a more general model, globally describing a link between protein structure and function as a “protein structure–function continuum,” where a given protein exists as a dynamic conformational ensemble containing multiple proteoforms (conformational/basic, inducible/modified, and functioning) characterized by a broad spectrum of structural features and possessing various functional potentials. The idea of various types of proteoforms is illustrated below, using human p53 as a case study. 

## 4. Multifunctionality of p53 and Intrinsic Disorder

Cellular tumor antigen p53 acts as a tumor suppressor. Its functions include inducing growth arrest [93] and apoptosis [94], which makes it a negative regulator of cell cycle progression. It is estimated that p53 mutations are involved in approximately 60% of human cancers [95]. Because of its ability to induce apoptosis, cell-cycle arrest, or DNA repair, p53 is widely considered to be a tumor suppressor that serves as a guardian of the genome [96]. However, there are many other cellular processes, e.g., cell differentiation and cell senescence, where p53 is known to play important roles [97]. Alternative initiation of translation, alternative promoter usage, and alternative splicing of the *TP53* gene generate multiple isoforms of the p53 protein, often with antagonistic functions [98]. This protein is known to have four structural/functional domains, an intrinsically disordered N-terminal transactivation domain, an ordered DNA binding domain located in the central part of this protein, and an intrinsically disordered C-terminal domain with tetramerization and regulatory functions [30,51]. 

**Figure 3:**
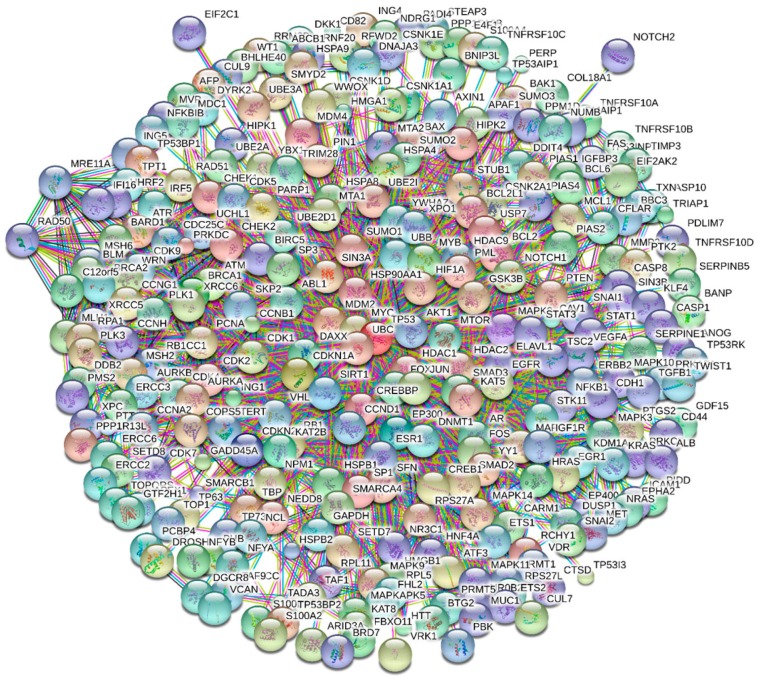
Analysis of the interactivity of human p53 (UniProt ID: by STRING computational platform that produces the network of predicted associations centered at the query protein [100]). Seven types of evidence are used to build the corresponding network, where they are indicated by differently colored lines: a green line represents neighborhood evidence; a red line—the presence of fusion evidence; a purple line—experimental evidence; a blue line—co-occurrence evidence; a light blue line—database evidence; a yellow line—text mining evidence; and a black line—co-expression evidence [100]. In this analysis, the most stringent criteria were used for the selection of interacting proteins by choosing the highest cutoff of 0.95 as the minimal required confidence level.

At the N-terminal transactivation domain, p53 interacts with many proteins, such as CBP/p300, CSN5/Jab1, Mdm2, RPA, TFIID, and TFIIH, to name a few [99], whereas the C-terminal regulatory domain of this protein is used for binding to 14-3-3, GSK3β, hGcn5, PARP-1, S100B(ββ), TAF, TAF1, TRRAP, and many other proteins [99]. These lists of interactors represent just the tip of the iceberg, since according to the different databases p53 can be engaged in interaction with almost 1000 partners. In fact, 990 interactions are reported in BioGrid, 411 interactions are shown in IntAct, N interactions are in MINT, and Y interactions can be found in DIP. This idea is illustrated by Figure 3, where a STRING-derived interactome of p53 is shown. This interactome was built using the highest confidence of 0.95 and it includes 302 nodes and 1884 edges, clearly illustrating exceptional binding promiscuity of p53. Therefore, consideration of intrinsic disorder and binding-induced folding can add value to the pathway analysis.

Among the central biological roles of p53 is the involvement of this transcription factor in the control of cell cycle and apoptosis via the transcriptional regulation of the expression of corresponding genes [101]. As a result, cancer development is often promoted by the loss of p53 function [102]. Therefore, it is not surprising to find that the interaction between the p53 and Mdm2, which is a E3 ubiquitin-protein ligase that inactivates p53 [103], is one of the most extensively studied protein–protein interactions, with almost 6000 PubMed publications (as of 2 November 2016) dedicated to this subject. It is established now that Mdm2 utilizes at least three different approaches to prevent p53 from activating its target genes [104]. These means are: (i) direct prevention of p53 interaction with transcription factors via Mdm2 binding to the p53 transactivation domain [105]; (ii) Mdm2-driven ubiquitination of p53 that targets this protein for proteasomal degradation [106]; and (iii) indirect prevention of p53 from activating genes via preferential export of the p53–Mdm2 complex from the nucleus due to the presence of a nuclear export signal in Mdm2 [107]. The X-ray crystallographic analysis of the p53–Mdm2 complex structure revealed that the Mdm2 surface possesses a deep groove, to which an N-terminal region of p53 (residues 13–29) binds in an α-helical form [108]. It is recognized now that the formation of this complex represents an important disorder-to-order transition, since, in its unbound form, the N-terminal region of p53 was shown to be highly flexible, lack fixed structure, but transiently form flexible amphipathic helical structure resembling α-helix that binds to Mdm2 [109]. 

## 5. Conformational/Basic Proteoforms of p53

Human p53 is a 393 residue-long protein, which has three functional/structural domains, the N-terminal region (residues 1–92), the central DNA-binding domain (DBD; residues 94–292), and the C-terminal region (residues 293–393) [110]. The N-terminal region contains three functional domains, transactivation domain 1 (TAD1; residues 1–40), TAD2 (residues 40–60), and a proline-rich region (PRR; residues 64–92). The C-terminal region is subdivided into a tetramerization or oligomerization domain (OD; residues 325–356), and a regulatory C-terminal domain (CTD; residues 356–393) [110,111]. The functional state of p53 is a homo-tetramer formed via a set of specific interactions between its ODs. The DBD domain of p53 is known to be characterized by high evolutionary conservation, whereas both N- and C-termini are much less conserved. Proper functionality of the p53 DBD critically depends on conservation of this domain since ~90% of cancer-related *P53* gene mutations are missense mutations in the DBD, resulting in the loss of DNA binding and hence affecting p53 function in cell cycle control [112].

**Figure 4:**
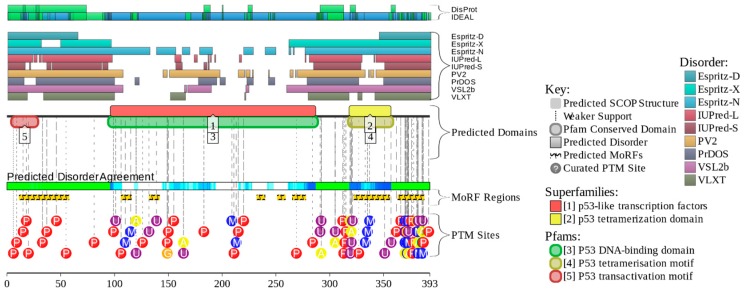
Intrinsic disorder propensity and some important disorder-related functional information generated for human p53 by the D^2^P^2^ database (http://d2p2.pro/) [113]. Here, the green-and-white bar in the middle of the plot shows the predicted disorder agreement between nine predictors, with green parts corresponding to disordered regions by consensus. Yellow bars show the location of the predicted disorder-based binding sites (molecular recognition features, MoRFs), whereas colored circles at the bottom of the plot show the location of various PTMs (P, phosphorylation; U, ubiquitination; M, methylation; A, acetylation; and G, glycosylation).

**Figure 5:**
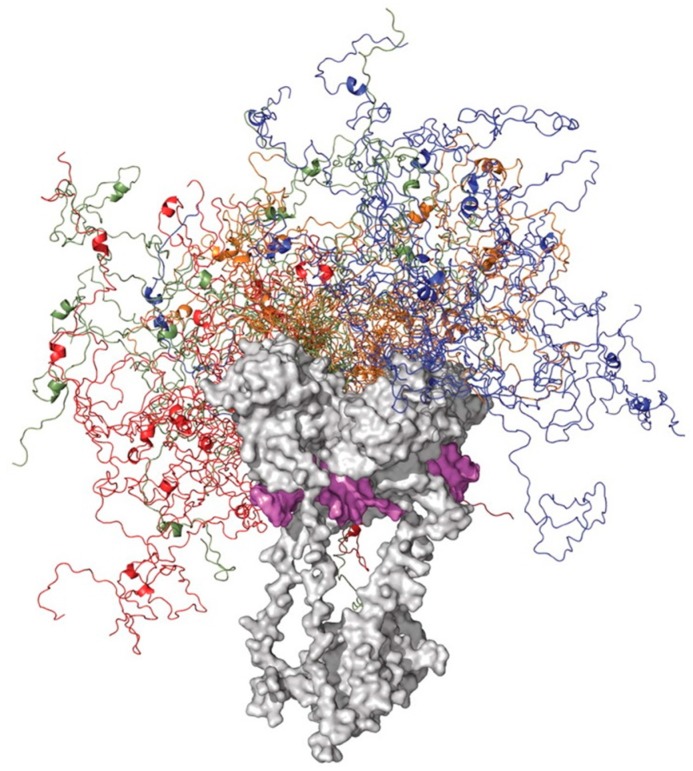
Modeled structure of the DNA-bound 1–360 region of p53. Here, the average ensemble structure of the N-terminal region (residues 1–95) is grafted to the DNA-bound p53_96–360_ region, where the p53_96–360_ (gray) and DNA (magenta) are shown in space-fill mode, and the N-terminal domains forming the four different monomers are shown in different colors for clarity. Twenty members of the conformational ensemble are shown for each monomer. This image is reproduced from the reference [121].

Despite serious efforts of multiple research groups, no experimentally derived structural information is available for the full-length p53 as of yet. This is because this protein represents a real challenge for structural biology, being too disordered for crystallography and too large (and tetrameric) for NMR analysis. In fact, Figure 4 shows that a very significant part of p53 is intrinsically disordered. This is illustrated by the plot generated by the D^2^P^2^ platform (http://d2p2.pro/) [113], which represents the outputs of IUPred [114], PONDR^®^ VLXT [115], PrDOS [116], PONDR^®^ VSL2B [117,118], PV2 [113], and ESpritz [119]. Figure 4 also shows that intrinsic disorder is crucial for function of this protein, since its numerous posttranslational modifications of different natures are preferentially located within the disordered regions and since it has multiple disorder-based protein binding sites. To further illustrate the overall structural complexity and exceptional conformational dynamics of this protein, Figure 5 represents a model generated by the superposition of the structure of the p53_96–360_ region that includes the DBD, linker, and OD in complex with DNA (determined by EM and SAXS [120]) with the average ensemble structure of the intrinsically disordered N-terminal region (generated using residual dipolar couplings (RDCs) from NMR spectroscopy and small-angle X-ray scattering (SAXS)) [98]. Although the disordered C-terminal region is not shown here, this image serves as an impressive illustration of the conformational (or basic or intrinsic) proteoform concept, where the protein exists as a dynamic conformational ensemble, members of which have rather different structures and therefore can be used for different functions.

## 6. Functioning Proteoforms of p53

Table S1 illustrates the continuous interest of the community of structural biologists in this protein by listing all p53-related entries currently found in the protein databank (PDB). It shows that the deposited structures can be grouped in several categories, such as p53 fragments bound to natural partners (proteins or DNA), various cancer-related mutant forms of the DBD, DBD (either wild type or containing disease-related mutations) bound to small molecules, and the tetrameric form of the wild-type OD and its several mutants designed to generate dimeric form. This multitude of complexes of different p53 fragments bound to various proteins can serve as an illustration of the functioning proteoform concept, where the original conformational ensemble is perturbed by function and interaction with the specific partners, with each partner able to induce unique and specific perturbations in the said ensemble. Sections below consider illustrative members of the functioning proteoform (various p53 fragments bound to different partners), which are grouped according to their positions within the p53 amino acid sequence and show members of the N-terminal proteoform, the DBD/OD proteoform, and the C-terminal proteoform. This section is intended to deliver an important message, namely, that the interactions with different binding partners or self-oligomerization can differently affect the structural properties of an IDP, giving rise to the partner-specific perturbations in its conformational ensemble and thereby creating different functioning proteoforms.

### 6.1. N-Terminal Proteoforms

**Figure 6:**
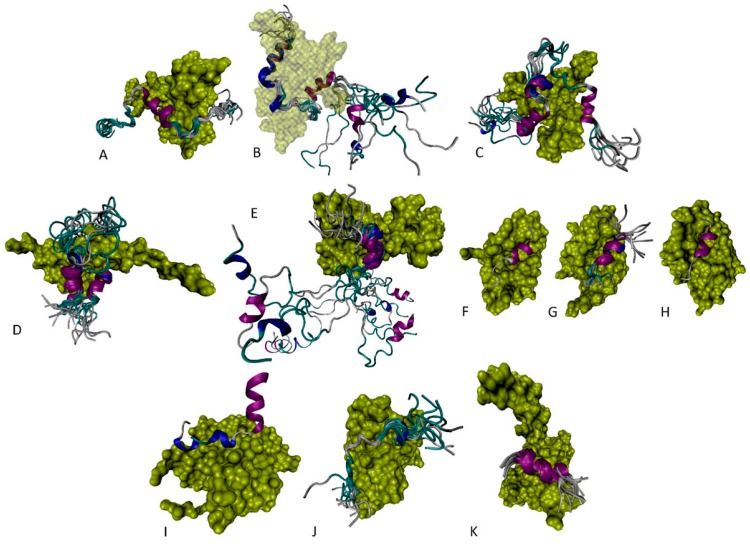
Complexes of the p53 fragments derived from its N-terminal region bound to different partners. Here, the first row of structures represents complexes involved: (**A**) 1–31 (PDB ID: 2K8F)); (**B**) 1–61 (PDB ID: 5HOU; and (**C**) 2–61 (PDB ID: 5HPD) fragments. The second row has complexes of the following p53 fragments: (**D**) 13–61 (PDB ID: 2L14); (**E**) 14–60 (PDB ID: 2LY4); (**F**) 15–29 (PDB ID: 1YCR); (**G**) 15–29 (PDB ID: 2MWY); and (**H**) 17–37 (PDB ID: 3DAC). The third row represents complexes involving the (**I**) 33–60 (PDB ID: 2B3G); (**J**) 41–62 (PDB ID: 2RUK); and (**K**) 45–58 (PDB ID: 2GS0) fragments of p53. Structures of binding partners are shown as yellow surfaces, whereas structures of p53 fragments are shown as ribbons colored according to the secondary structure content. In panel **B**, structure of binding partner (TAZ1 domain of CBP) is shown in a semi-transparent form to simplify visualization of the position of the p53 polypeptide chain within the p53-TAZ1 complex.

Figure 6 collects several complexes of the various fragments derived from the N-terminal region of p53 (the region that looks like a set of decorated noodles in Figure 5). Figure 6A represents a solution structure of the complex between the N-terminal half of the p53 TAD (residues 1–31) and the transcriptional adapter zinc finger domain TAZ2 of p300 (residues 1723–1812 in the UniProt ID: Q09472) and shows that, upon binding to this partner, TAD forms a short α-helix (residues 17–24) surrounded by highly flexibile N- and C-terminal regions (residues 1–16 and 25–31) [122]. Although the solution NMR structure was solved for the complex containing both a p53 TAD region and a p300 TAZ2 domain, representation of the ordered TAZ2 domain structure in this image is limited to showing just one member of the TAZ2 conformational ensemble (see yellow surfaces). 

The next two images in Figure 6 show the NMR structures of specifically designed fusions of the N-terminal transactivation domain (TAD) of p53 (residues 1–61 or 2–61) with the TAZ1 or TAZ2 domains of the CREB-binding protein, CBP (Figure 6B,C, respectively) [123]. Similar to the TAD-p300_TAZ2_ situation, structures of the CPB TAZ1 and TAZ2 domains are shown for just one illustrative member of the corresponding TAZ1 or TAZ2 conformational ensemble; this is because the structures of these domains in the fusion constructs are well-defined and similar to the structures in their un-bound states [123]. Figure 6B,C show that the p53 TAD region binds to different targets using a bipartite mode [123], for which TAD evolved to have two interaction motifs, AD1 (residues 18–26) and AD2 (residues 44–54). Although these regions are mostly disordered in the unbound state, upon binding to TAZ1 or TAZ2 they are able to fold into short amphipathic α-helices. The remaining parts of the p53 TAD, i.e., its N-terminal tail (residues 1–17), the linker between AD1 and AD2 (residues 27–43), and the C-terminal tail (residues 55–61), remain flexible. 

Since this analysis revealed that AD1 and AD2 binding to the target protein is synergistic, it was pointed out that caution should be used in the analysis and interpretation of functional and structural studies conducted with the isolated motifs [123]. The overall configuration of the bound form of TAD, as well as the length of the induced AD1 and AD2 helices, depend on the binding partner. For example, although AD2 motif binds to both TAZ1 and TAZ2 in a hydrophobic groove at the interface between the α1, α2, and α3 helices, the AD2 helix is oppositely oriented in the two structures [123]. Figure 6D shows the structure of the solution NMR structure of a complex between the TAD 13–61 fragment of human p53 and the nuclear receptor coactivator binding domain of CBP (residues 2061–2117 in UniProt ID: P45481) [124]. Although this p53 TAD fragment contains both binding motifs AD1 (residues 19–26) and AD2 (47–53) that form short α-helices, the lengths of these helices (especially AD2) are noticeably shorter than their lengths in other complexes, and the overall configuration of the TAD bound form is profoundly different from those reported for the p53 TAD bound to the CPB TAZ1 or TAZ2 domains.

Curiously, Figure 6E illustrates that the bipartite interaction is not the only binding mode used by the p53 TAD, since in its complex with the A-box (2–84 fragment) of the high mobility group protein B1 (HMGB1; UniProt ID: P09429) it uses a single helical turn (residues 41–44) connected by a sharp turn (residues 45 and 46) to a longer helix (residues 47–55) [125]. Note that this structure of bound AD2 is rather different from structures of AD2 bound to CBP and p300. Also, despite the fact that the 1–93 region of p53 was used in structural analysis, the structure is reported only for the 14–60 fragment of p53 [125]. 

Plots F, G, and H in Figure 6 show the structures of the p53 AD1 bound to the N-terminal domains of human Mdm2 (residues 17–125; UniProt ID: Q00987; Figure 6F) [108], human Mdm4 (residues 23–111; UniProt ID: O15151, Figure 6G) [126], and Mdm4 from *Danio rerio* (residues 15–129; UniProt ID: Q7ZUW7, Figure 6H) [127]. In these structures, the helical region induced in the bound forms of AD1 (residues 19–24, 18–24, and 19–24, respectively) is noticeably shorter than that discussed in previous sections, providing further support to the notion that p53 TAD binding to its partners might require the entire domain instead of being synergistic; it was pointed out that caution should be used in the analysis and interpretation of functional and structural studies conducted with the isolated motifs [123].

Figure 6I represents the crystal structure of a complex between the AD2-containing fragment of p53 (residues 33–60) and the 70 kDa DNA-binding subunit of the replication protein A (RPA70N; residues 1–120; UniProt ID: P04637). In this complex, in addition to the α-helical motif (residues 47–55) TAD fragment folds into two short amphipathic 3_10_ helices, residues 36–38 and 41–44 [128]. The NMR solution structure of the similar fragment of p53 (residues 41–62) bound to the pleckstrin homology (PH) domain of the general transcription factor IIH subunit 1 (TFIIH p62, residues 1–108 in UniProt ID: P32780) is presented in Figure 6J, which clearly shows that this region can bind to its partners without gaining significant helical structure [129]. 

Finally, Figure 6J represents the NMR solution structure of the complex between the AD2 (residues 45–58) and the N-terminal pleckstrin homology PH domain of the p62/Tfb1 subunit of human RNA polymerase II transcription factor B (residues 1–115; UniProt ID: P32776) [130], where AD2 adopts a well-developed helical structure. 

### 6.2. DBD/OD Proteoforms

**Figure 7:**
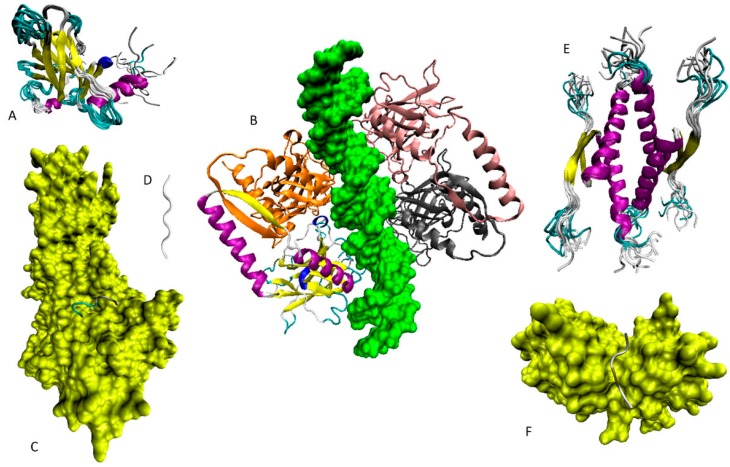
Complexes of the p53 fragments derived from its central region bound to different partners: (**A**) 94–297 (PDB ID: 2FEJ); (**B**) 94–388 (PDB ID: 4MZR); (**C**) 144–154 (PDB ID: 2YDR); (**D**) 252–258 (PDB ID: 4RP6); (**E**) 319–360 (PDB ID: 1OLH); and (**F**) 358–363 (PDB ID: 2FOO). The structures of protein-binding partners are shown as yellow surfaces; the structure of DNA is shown as green surface; whereas structures of p53 fragments are shown as ribbons colored according to the secondary structure content.

Figure 4 and Figure 5 show that the central region of human p53 containing DBD and OD is characterized by the presence of a substantial amount of ordered structure. In agreement with these observations, Figure 7A,B shows the NMR solution structure of the DBD [131] and the crystal structure of the complex between the mutant form of the p53 central region containing PDB and OD and DNA [132]. 

In addition to the structure of DBD alone (wild-type and numerous cancer-related mutant) and various complexes of this domain with DNA, structural information is available for the complexes of the p53 DBD bound to several protein partners [30]. 

Peculiarities of the structural perturbations induced in the ordered DBD by interaction with different partners (DNA [133], 53BP1 [134], 53BP2 [135], and the large-T antigen (LTag) from simian virus 40 [136]) were analyzed in the dedicated study, where it was pointed out that “multiple partners of p53 are accommodated by reusing similar binding interfaces. This is facilitated by small scale or large scale structural differences, which range from differences in side chain conformation to backbone rearrangements” [30]. In other words, the ability of DBD to be engaged in multiple interactions with several binding partners relies mostly on some local structural adjustments. 

Figure 7C shows the 144–154 fragment of p53 co-crystallized with the N-terminal region of the *O*-GlcNAcase containing catalytic domain (residues 31–618, UniProt ID: Q0TR53) [137]. The analyzed regions of p53 contains a natural glycosylation site (Ser149). Although this 144–154 fragment is a part of the ordered DBD, it is located in the solvent-accessible loop and therefore can be engaged in interaction with binding partners. Figure 7D shows the crystal structure of a short p53 fragment involved in the formation of amyloid fibrils [138]. Although the isolated fragment is in the extended conformation (see Figure 7D), within the context of the DBD, it is present as one of the β-strands. The solution structure of the oligomerization domain (residues 319–360) is shown in Figure 7E [139]. The structure of the OD was described as follows: “The domain forms a 20-kilodalton symmetric tetramer with a topology made up from a dimer of dimers. The two primary dimers each comprise two antiparallel helices linked by an antiparallel beta sheet. One beta strand and one helix are contributed from each monomer. The interface between the two dimers forming the tetramer is mediated solely by helix-helix contacts. The overall result is a symmetric, four-helix bundle with adjacent helices oriented antiparallel to each other and with the two antiparallel beta sheets located on opposing faces of the molecule.” [139]. Although this domain is structured, it folds during the tetramerization process and the shown structure is acquired upon the formation of the complex [30]. Finally, Figure 7F shows a crystal structure of the complex formed between the 358–363 fragment of p53 and the N-terminal domain of the ubiquitin carboxyl-terminal hydrolase 7 HAUSP/USP7 (residues 51–205; UniProt ID: Q93009) [140]. This fragment of p53 is a part of the linker region connecting OD and CBD and it is disordered in the unbound form and gains an irregular structure during interaction with its partner.

### 6.3. C-Terminal Proteoforms

**Figure 8:**
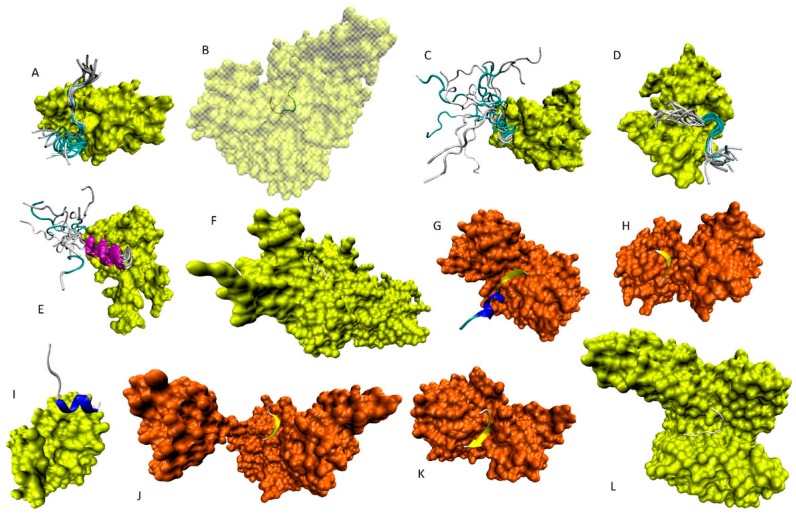
Complexes of the p53 fragments derived from its C-terminal region and bound to different partners. Here, the first row of structures represents complexes involved: (**A**) 363–377 (PDB ID: 2MWO); (**B**) 365–377 (PDB ID: 3TG5); (**C**) 367–386 (PDB ID: 1JSP); and (**D**) 367–387 (PDB ID: 2MWP), fragments; The second row has complexes of the following p53 fragments: (**E**) 367–388 (PDB ID: 1DT7); (**F**) 369–377 (PDB ID: 1XQH); (**G**) 372–389 (PDB ID: 1YC5); and (**H**) 376–388 (PDB ID: 4BV2); The third row represents complexes involving the (**I**) 377–386 (PDB ID: 4X34); (**J**) 379–383 (PDB ID: 4ZZJ); (**K**) 379–386 (PDB ID: 4BUZ); and (**L**) 385–393 (PDB ID: 3LW1) fragments of p53. Structures of protein-binding partners are shown as yellow or orange surfaces, whereas structures of p53 fragments are shown as ribbons colored according to the secondary structure content. In panel **B**, structure of binding partner (*N*-lysine methyltransferase SMYD2) is shown in a semi-transparent form to simplify visualization of the position of the p53 polypeptide chain within the corresponding complex.

Figure 8 provides a structural description of the C-terminal proteoform by showing solution or X-ray structures of different p53 fragments derived from its negative regulatory C-terminal domain (CTD; residues 364–393). 

Figure 8 shows that the N-terminal part of this region can be folded either into irregular structure, configuration, and conformational dynamics that dramatically depend on a binding partner (as in the complexes of 363/369–377/388 peptide with the Tudor-like region of the tumor suppressor p53-binding protein 1 (P53BP1, residues 1484–1603; UniProt ID: Q12888; Figure 8A,D) [141]; *N*-lysine methyltransferase SMYD2 (UniProt ID: Q9NRG4; Figure 8B) [142]; CBP bromodomain (residues 1081–1197; UniProt ID: Q92793; Figure 8C) [143]; H3 lysine-4 specific histone-lysine *N*-methyltransferase (residues 107–366; UniProt ID: Q8WTS6; Figure 8F) [144]) or have significant α-helical structure (as in the case of the complex between the p53 367–388 fragment and calcium-bound S100ββ (UniProt ID: P04631; Figure 8E) [145]).

Similarly, the central region of the CTD (residues 372/379–386/389) is also characterized by a remarkable structural diversity in the bound state and can be found in helical/β-structural form, as in the complex of the 372–389 peptide with the NAD-dependent deacetylase Sir2 from *Thermotoga maritima* (UniProt ID: Q9WYW0; Figure 8G) [146], or can be helical, as in the complex with the Tudor-like domain of the P53BP1 (residues 1484–1603; UniProt ID: Q12888; Figure 8I) [147], or be in a β-structural or extended configuration, as in the complexes between the 376–388 fragment and the NAD-dependent deacetylase Sir2 from *Thermotoga maritima* (UniProt ID: Q9WYW0; Figure 8H) [148], the 379–383 fragment and the NAD-dependent protein deacetylase sirtuin-1 (residues 183–503; UniProt ID: Q96EB6; Figure 8J) [149], and the 379–386 fragment and the NAD-dependent deacetylase Sir2 from *Thermotoga maritima* (UniProt ID: Q9WYW0; Figure 8K) [148]. Figure 8L closes this gallery of the functioning p53 proteoforms by showing the crystal structure of the complex of the very C-terminal p53 fragment (residues 385–393) and the 14-3-3 protein sigma (UniProt ID: P31947) [150].

### 6.4. Tetrameric Proteoforms

The biologically active form of p53 (i.e., the form in which this protein binds to DNA) is a homo-tetramer. Besides its importance for the site-specific DNA binding [151], homo-tetramer formation of p53, which is mediated by the C-terminal tetramerization domain of this protein, is essential for protein–protein interactions and may play a role in posttranslational modifications [152]. One should keep in mind that tetramerization can be affected by PTMs, alternative splicing, or cancer-related mutations, and various hetero-tetramers can be present, whose protomers could be wild-type or mutants of the canonical p53, or its alternatively spliced isoforms. Individual protomers can also possess different PTMs or be unmodified. 

Furthermore, several protein–protein interactions of p53 are regulated by its tetramerization. For example, the tetramerization domain of p53 is required for interaction of this protein with the member of the BCL2 family, Bcl-2 homologous antagonist/killer (BAK) and is needed for the efficient oligomerization of this important pro-apoptotic protein [153]. Furthermore, several proteins were shown to bind directly to the oligomerization domain of p53, whereas the interaction of other proteins with p53 was shown to be dependent on the oligomeric status of this protein [154,155,156,157]. Finally, tetramerization plays a crucial role in the cellular localization of p53, since tetramer formation blocks a leucine-rich nuclear export signal (NES) located in the oligomerization domain and thereby prevents the nuclear export of p53 [158]. All these factors further increase the complexity of the tetrameric p53 and generate a very wide array of functioning proteoforms.

## 7. Inducible/Modified Proteoforms of p53

### 7.1. Alternative Splicing-Induced p53 Proteoforms

As was already noted, due to alternative splicing, alternative promoter usage, and alternative initiation of translation, the *TP53* gene, which is composed of 11 exons, can be expressed as a set of multiple isoforms [98,159]. A brief description of the nine most common isoforms is given below. The canonical p53 protein (p53α or p53) has a sequence of 393 residues that includes the full TAD sequence and the longest C-terminal domain. Alternative splicing affecting the C-terminal domain generates two C-terminally truncated isoforms, p53β and p53γ, which differ from the canonical isoform by possessing changes within their 332–341 (IRGRERFEMF → DQTSFQKENC) or 332–346 regions (IRGRERFEMFRELNE → MLLDLRWCYFLINSS), and by missing the 332–393 or 347–393 regions, respectively. Because of the use of the alternative translation initiation sites or alternative splicing, three different ΔN variants missing different parts of the N-terminal domain (Δ40p53α, Δ133p53α, and Δ160p53α) can be produced in addition to the canonical p53α isoform. The use of the same alternative translation initiation sites within the frames of the p53β and p53γ isoforms generates six more p53 splice variants, Δ40p53β, Δ40p53γ, Δ133p53β, Δ133p53γ, Δ160p53β, and Δ160p53γ. 

It is known that splice variants of the human *TP53* gene can be differentially expressed in human tumors compared with normal tissue [160], and are also differentially expressed in different tumors [161]. Importantly, different p53 splice variants are not only characterized by different expression levels in different cancer types but also possess different biological functions and can even affect each other’s activities [159]. For example, the p53β isoform can differentially bind to the p53-inducible promoters and specifically enhances p53 transcriptional activity on the BAX promoter but not on the p21 promoter [161]. It is also characterized by reduced pro-apoptotic activity compared with p53α [161]. The p53β isoform is expressed in most normal tissue except for the brain, fetal brain, fetal liver, lung, muscle, prostate, and spinal cord [161]. The p53γ variant can enhance transcriptional activity only on the BAX promoter [162]. The p53γ variant, being expressed in most normal tissues, was not detected in the fetal brain, fetal liver, lung, spinal cord, spleen, and testes [161].

It was pointed out that, as a response to stress stimuli, p53α can bind and transactivate the internal *TP53* promoter, thereby controlling the expression of its own isoform, Δ133p53α [162]. The expression of various genes can be regulated by Δ133p53α–p53α interactions leading to the inhibition of apoptosis, G1 cell-cycle arrest, and replicative senescence [163]. These same Δ133p53α–p53α interactions can also enhance blood vessel formation, endothelial cell migration, and metastasis formation [163]. 

Δ133p53, Δ133p53β, and Δ133p53γ isoforms generated by the use of the alternative promoters in intron 4 and alternative splicing of intron 9 were shown to have different tissue distributions [161]. Here, the Δ133p53 variant was found in most normal tissue except for breast, prostate, skeletal muscle, and uterus; the Δ133p53γ isoform was expressed in most normal tissue except for the brain, breast, fetal liver, heart, intestine, lung, and salivary gland, whereas the Δ133p53β variant was preferentially expressed in the bone marrow, colon, fetal brain, intestine, and testes [161]. Also, differences in the cellular localization of the p53 isoform were reported, with the canonical p53 form being exclusively localized in the nucleus; Δ133p53 and p53β were preferentially localized in the nucleus but also found in the cytoplasm, and p53γ shuttled between the nucleus and the cytoplasm [161]. It was also pointed out that the Δ133p53 proteoform that lacks the N-terminal 133 residues and is generated by the alternative promoter usage, and the p53β proteoform that carries out an alternative C-terminus and is produced due to the alternative splicing, can be involved in controlling cellular senescence, which is characterized by the irreversible stop in proliferation of the metabolically active cells [164].

The Δ40p53 isoforms (also named N-terminally truncated p53 protein (p53/47), or ΔNp53) are generated by alternative splicing of intron 2 and/or alternative initiation of translation [165]. Since these isoforms lack the first 39 amino acids, they do not have TAD1, but retain TAD2. The lack of TAD1 precludes p53/47 from interaction with Mdm2. As a result, properties of the total cellular pool of the p53 proteoforms can be changed. In fact, even in the presence of Mdm2, the total cellular levels of p53 are stabilized due the increased expression of p53/47, and the expression levels of the p53-induced genes are changed, reflecting the importance of the ratio of full-length p53 to p53/47 for the regulation of the biological activities of p53 [166]. Furthermore, p53/47 was shown to be related to the control of folding, oligomerization, and PTM status of the p53 tetramers [167]. It is also able to diversify the biological activity of p53 in a cell stress-dependent manner [167]. For example, it was shown that under the stress conditions induced by the treatment of cells with the DNA damaging drug doxorubicin the p53/47 was recruited to the p21 promoter, causing an 18-fold increase in the expression level of this protein [167]. Endoplasmic reticulum (ER) stress enhanced the p53/47 mRNA translation and caused formation of p53/47 homo-tetramers, leading to G_2_ cell-cycle arrest without affecting G_1_ progression. This was in contrast to homo-oligomers of the full-length p53 that are known to promote G_1_ arrest but have no effect on the G_2_ [168]. 

Summarizing, the Δ40p53α isoform cannot interact with Mdm2, is characterized by the impaired transcriptional activation capability, and can negatively regulate the transcriptional and growth-suppressive activities of p53α by oligomerizing with the canonical isoform [169]. Curiously, p53α was shown to be protected from degradation by the Mdm2 pathway when co-transfected with Δ40p53 [170]. It is also likely that the Δ40p53 isoform is involved in the control of entry in the S phase since the Δ40p53/p53α ration was shown to vary during the cell cycle [169]. 

Little is currently know about the functionality of Δ40p53β and Δ40p53γ. Finally, the Δ160p53α, Δ160p53β, and Δ160p53γ isoforms are characterized by a lack of the first 159 amino acids [165]. Although the currently available information about these p53 variants is very limited, it was found that this group of p53 isoforms was expressed in K562 cells, which were originally considered “p53-null” cells [171,172]. Also, it was proposed that Δ160p53β may have a role in erythroid differentiation [173]. 

In short, different mechanisms of gene expression control are used in the cell for the regulation of levels of different p53 isoforms possessing different biological functions. Therefore, it seems that controlled production of different alternative proteoforms helps the cell to differentiate between p53 activation and the response to diverse stresses.

Although the involvement of p53 in cancer pathogenesis is typically reflected in the presence of numerous mutants (see below), some types of cancer, such as AML or breast cancer, do not have frequent p53 mutations [165]. Based on the observation that different p53 isoforms can be differentially expressed in tumors compared with normal tissue, an importance of alternative isoforms in carcinogenesis became clear [174,175,176]. For example, abnormal expression of p53 isoforms was reported in acute myeloid leukemia (AML), cholangiocarcinoma, colon carcinoma, glioblastoma, head and neck tumors, lung tumors, and ovarian tumors [177,178,179,180,181,182]. It was shown that normal breast tissue expresss p53α, p53β, and p53γ, whereas the expression of p53β and p53γ is lost in 60% of breast tumors, which instead frequently overexpress the isoform Δ133p53 [183]. The p53β and Δ40p53 isoforms were shown to be expressed in melanoma cells but not in melanocytes or fibroblasts [184], whereas renal cell carcinoma (RCC) was shown to be characterized by the overexpression of the p53β and Δ133p53 isoforms in comparison with normal cells [185,186]. 

**Figure 9:**
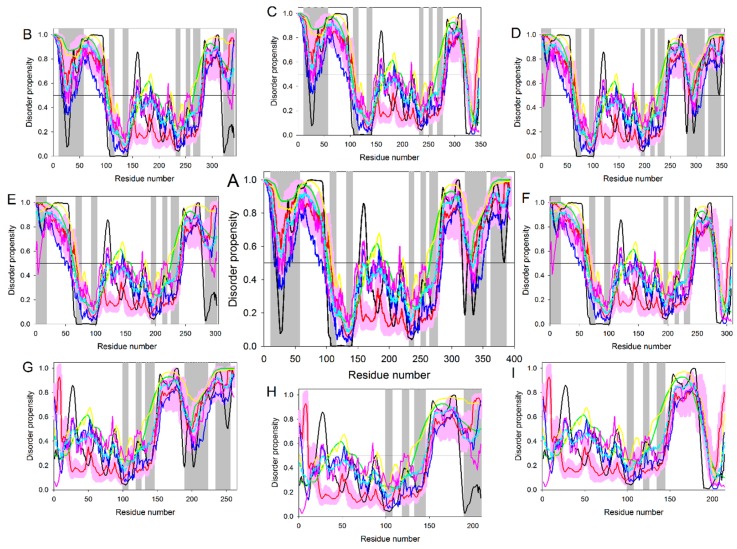
Analyzing intrinsic disorder in various alternatively spliced variants of human p53: p53α (**A**); p53β (**B**); p53γ (**C**); Δ40p53α (**D**); Δ40p53β (**E**); Δ40p53γ (**F**); Δ133p53α (**G**); Δ133p53β (**H**); and Δ133p53γ (**I**). Disorder profiles were generated using four algorithms from the PONDR family, PONDR^®^ VLXT (black curves), PONDR-FIT (red curves), PONDR^®^ VL3 (green curves), and PONDR^®^ VSL2 (yellow curves) [115,117,118,187,188,189], as well as the IUPred web server for predicting short (blue curves) and long disordered regions (pink curves) [114]. Bold cyan dashed lines show the mean disorder propensities calculated by averaging disorder profiles of individual predictors. The light pink shadow around the PONDR^®^ FIT shows the error distribution. In these analyses, predicted intrinsic disorder scores above 0.5 are considered to correspond to disordered residues/regions. Positions of the disorder-based binding sites (molecular recognition features, MoRFs) found by the ANCHOR algorithm [190,191] are shown as gray shaded areas.

Figure 9 represents an intrinsic disorder-based view of the p53 proteoforms generated by alternative splicing, alternative promoter usage, and alternative initiation of translation. In agreement with the previously reported observations that alternative splicing primarily affects regions of mRNA encoding for the IDPRs in proteins [48], Figure 9 shows that disorder profiles of p53 are dramatically affected by alternative splicing. Since various isoforms of p53 are generated by combinatorial elimination of intrinsically disordered N- and C-terminal regions, the resulting variants are characterized by a decreasing amount of disorder in comparison with the full-length or canonical form. Also, because the N- and C-terminal domains of p53α are regulatory regions involved in binding to a multitude of partners, their removal results in the elimination of numerous protein–protein interactions. It is important to remember that the overall functionality of p53 and its involvement in carcinogenesis may rely on precise control and changes in the relative contents of various alternative isoforms. 

The fact that different alternative variants of p53 are differently distributed among tissues is in agreement with the previously reported observation that among the alternatively spliced exons of many proteins there are tissue-specific exons that are crucial for tissue identity maintenance [192]. Furthermore, such tissue-specific protein segments are enriched in IDPRs, have numerous sites of various PTMs, contain disorder-based binding motifs, and are common for signaling, development, and disease-related proteins [192]. All this indicates that the alternative splicing-generated proteoforms could alter protein “function in different tissues and organisms by rewiring interaction networks through the recruitment of distinct interaction partners via the alternatively spliced disordered segments” [193]. It was also pointed out that in addition to chromosomal translocations, altered expression, frustrated posttranslational modifications, deviant proteolytic degradation, and defective trafficking, aberrant alternative splicing serves as an important factor inducing pathogenic transformations of IDPs and generating abnormal proteoforms [194].

### 7.2. PTM-Induced p53 Proteoforms

**Figure 10:**
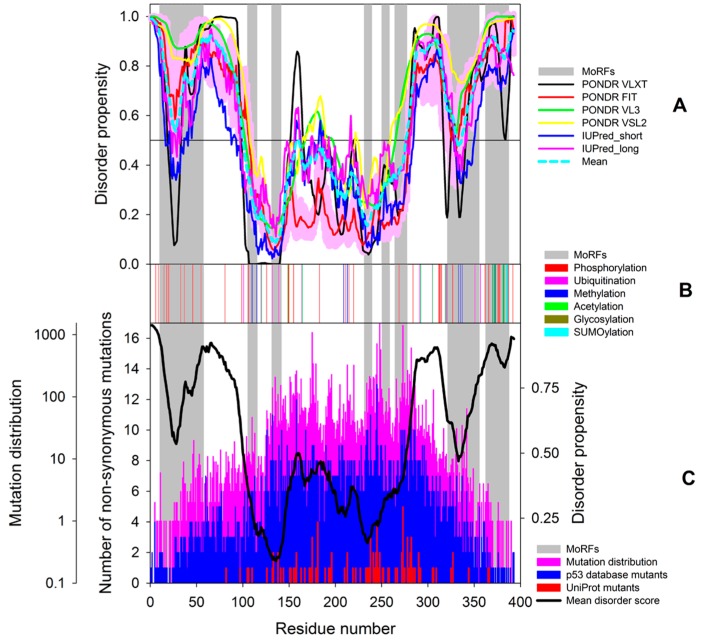
Distribution of intrinsic disorder propensity (**A**); PTM sites (**B**) and pathological mutations (**C**) within the sequence of human p53. In plot (**A**), disorder profiles are generated using PONDR^®^ VLXT, PONDR-FIT, PONDR^®^ VL3, PONDR^®^ VSL2, and IUPred. The bold cyan dashed line shows the mean disorder propensities calculated by averaging the disorder profiles of individual predictors. The light pink shadow around the PONDR^®^ FIT shows the error distribution. In all plots, the positions of disorder-based binding sites (molecular recognition features, MoRFs) found by the ANCHOR algorithm are shown as gray shaded areas. Plot (**C**) shows the sequence distribution of pathological mutations (pink bars; note the semi-logarithmic scale of this graph) and the abundance of different non-synonymous mutations (blue bars) in the UMD TP53 mutation database (http://p53.fr/), and distribution of pathological mutations annotated in UniProt (red bars).

Figure 4 and Figure 10B show that human p53 is heavily decorated by various PTMs, whereas Figure 4 and Figure 10A show that the vast majority of PTMs are located within IDPRs and can affect the MoRFs of this protein. In fact, it was pointed out that this protein can be modified at over 60 of its 393 residues that can have several different PTMs, such as acetylation, ADP ribosylation, cysteine and methionine oxidation, cysteine alkylation, glycosylation, methylation, NEDDYlation, nitration, *O*-GlcNAcylation, phosphorylation, poly-ribosylation, SUMOylation, and ubiquitination [152,195,196]. Importantly, the major p53 regions affected by PTMs are its intrinsically disordered N- and C-terminal regulatory domains (see Figure 4 and Figure 10). Therefore, various PTMs play a number of important roles in the regulation of p53 activity and tetramerization [152,195,196]. PTMs were pithily defined as “cooperative integrators of function” in p53 function since they appear in response to various stresses (genotoxic or non-genotoxic) and can trigger various subsequent events [195].

Furthermore, many p53 PTMs are interdependent and operate through multiple intertwined pathways and cooperative events, thereby generating complex and rather unexpected outcomes [195]. For example, ATM- and ATR-mediated phosphorylation of Ser15 in response to genotoxic stress triggers subsequent sequential modifications of many residues [197,198,199,200,201]. The protein kinase CK1-mediated phosphorylation of p53 at Thr18 requires prior phosphorylation of Ser15 [202]. ATM-mediated phosphorylation of p53 at Ser15 or Thr18, or other residues of the transactivation domain, promotes the subsequent recruitment of histone/lysine acetyltransferases (HATs) CBP and p300, leading to the acetylation of the p53 DBD and CTD at multiple lysine residues [122,203,204,205,206,207]. Although exposure of cells to UV light or ionizing radiation was shown to result in acetylation of human p53 at Lys382 and phosphorylation at Ser33 and Ser37 in vivo, differential inhibition of the p53 acetylation in vitro was reported for the N-terminal p53 peptides phosphorylated at Ser33 and/or at Ser37 [201]. 

The aforementioned examples provide an illustration of how the creation of one PTM-dependent proteoform can affect the formation of other proteoforms. Furthermore, PTMs can regulate p53’s interaction with its binding partners. For example, the affinity of the p53_1–39_ fragment of the p53 TAD1 for the TAZ2 domain of p300 was shown to be increased in response to the Ser15 or Thr18 mono-phosphorylation and was further increased by di-phosphorylation at Ser15–Ser37 or Thr18–Ser20 sites [203]. In a systematic analysis of the effect of phosphorylation on the p53 TAD1 interactivity, it was established that the affinity of this domain to CH3 and TAZ1 can be increased up to sevenfold due to the Thr18 phosphorylation, whereas phosphorylation of Ser15, Ser20, Ser33, Ser37, Ser46, and Thr55 causes smaller affinity increases. However, the efficiency of the p53 TAD binding to CH3 and TAZ1 was increased 40- and 80-fold as a result of the hepta-phosphorylation of all Ser and Thr residues [207]. On the other hand, the p53 TAD binding to Kix and IBiD was less sensitive to TAD1 phosphorylation [207], and phosphorylation of sites within TAD2 had negligible effects on p300 binding [203,207]. Also, phosphorylation of p53 at several N-terminal sites, such as Ser15, Thr18, and Ser20, was shown to block Mdm2 binding and lead to decreased p53 turnover [200,208,209,210,211,212]. The phosphorylation of p53 at Ser_6_ and Ser_9_ is needed for the efficient interaction with the TGF-β-activated Smad2 [195].

p53 acetylation is another PTM that is important for many biological functions of this protein [213]. Nine of the 11 acetylatable lysine residues of p53 are located within the CTD, and the remaining two (Lys1_20_ and Lys_163_) control functions of the DBD. Several lysine residues of p53 that can be ubiquitinated (or subjected to other PTMs) are also acetylated. In response to different types of stress, the acetylation levels of p53 are increased, which is associated with the activation of this protein, leading to the recruitment of various cofactors needed for its promoter specific transcriptional activity [214,215]. Acetylation of the DBD-located lysines generates different outputs, with Lys_120_ acetylation leading to the activation of genes related to apoptosis but not to cell cycle arrest, and with the acetylation of Lys_164_ resulting in activation of the majority of p53 target genes [213]. Importantly, ubiquitination and acetylation (as well as NEDDylation and methylation) are mutually exclusive events that have different outcomes for p53 regulation [195]. Therefore, acetylation of C-terminal lysine residues (Lys_305_, Lys_370_, Lys_372_, Lys_373_, Lys_381_, Lys_382_, and Lys_386_) and one DBD residue (Lys_164_) by CBP (KAT3A)/p300 (KAT3B) and acetylation of K320 by PCAF (KAT2B) represents a response to various types of stresses that lead to stabilization and activation of p53, likely due to the inability of Mdm2 to ubiquitinate the acetylated residues [195,196,214,215]. 

Polyubiquitination, ubiquitination, SUMOylation, and NEDDylation are important PTMs caused by the reversible covalent addition of proteins (ubiquitin, SUMO, or NEDD) to the specific lysine residues in a target protein. In p53, most lysine residues modified by ubiquitination (except to Lys_101_) are located within the DBD and the CTD, whereas all lysines that are NEDDylated (Lys_320_, Lys_321_, Lys_370_, Lys_372_, and Lys_373_) or SUMOylated (Lys_386_) are positioned inside the CTD [216]. The importance of these PTMs is determined by their influence on the fate of a modified protein: polyubiquitination is known to serve as a signal for the proteasomal degradation of a protein (and therefore is responsible for p53 deactivation), whereas monoubiquitination, NEDDylation, and SUMOylation may target p53 to different cellular locations [217]. 

Importantly, some p53 PTMs are known to be dependent on the oligomeric state of this protein. This includes the DNA damage-inducible p53 phosphorylation at Ser_20_ leading to the inhibition of p53–Mdm2 interaction [208], acetylation of the C-terminal Lys_382_ residue needed for enhancing the DNA-binding affinity of p53 [201,218], acetylation of the C-terminal Lys320 mediated by the p300 acetyltransferase that can only bind to the p53 tetramer [218], E3 ubiquitin ligase Pirh2-induced preferential ubiquitination of the tetrameric form of p53 [219], and Mdm2-mediated poly-ubiquitination of p53 [220]. Despite the fact that tetramerization seems to be important for p53 ubiquitination and poly-ubiquitination, the oligomeric state has little effect on its proteasome degradation of this protein [220]. 

There is no need to describe all known p53 PTMs and their outputs for function and dysfunction of this protein. Interested readers are encouraged to look at dedicated reviews, of which there are many (e.g., [97,195,221,222,223,224,225,226,227]). However, one should remember that PTMs clearly represent an important mechanism increasing the functional and structural heterogeneities of p53 proteoforms. Various modifications play different roles in the regulation of p53 stability, the efficiency of numerous p53 interactions with other proteins and DNA, and the control of the transcription factor activity of this protein needed for the balanced response to a wide spectrum of intrinsic and extrinsic stresses. Both normal PTMs and aberrant modifications of p53 may also play a number of roles in the development of pathological conditions. Also, since PTMs primarily happen within the IDPRs of p53 that serve as biding sites for >300 protein partners, the major mechanism by which PTMs control and modulate p53 activity is by moderating, modifying, and adjusting these interactions rather than by alteration of p53’s structure. Finally, all p53 PTMs are potentially reversible. 

### 7.3. Mutation-Induced p53 Proteoforms

The *TP53* gene located on chromosome 17p13.1 is one of the most frequently mutated genes in human cancers [102,228], with this gene being a subject to inactivation by mutation or deletion in >50% of sporadic cancers [229]. Mutations in the *TP53* gene are known to result in loss of p53 function, negative complementation, or gain of oncogenic function, thereby leading to the increase of tumorigenicity and invasiveness of cancer. Non-synonymous or missense substitutions can generate new codons that code for different amino acids. Other mutations (so-called indels that lead to the insertion or deletion of bases in DNA) can cause frame-shifts or abnormal splicing. The nonsense mutations lead to the appearance of a premature stop codon or incorporation of a nonsense codon in the transcribed mRNA, resulting in a truncated, incomplete, and usually nonfunctional protein. Cancerous p53 mutations can be of somatic (non-inherited de novo mutations that originate and accumulate in the cells during a lifetime and cause “sporadic” tumors) or germline origin (inherited mutations causing familial tumors). 

Somatic *TP53* mutations can be found in almost every type of cancer and are present in half of all ovarian, colorectal, and esophageal cancers [230,231]. Missense mutations are the most common somatic substitutions found in the *TP53* gene, accounting for ~77% of all somatic mutations. Germline mutations were reported for more than 660 families, where they cause Li–Fraumeni syndrome (LFS) and Li–Fraumeni-like syndrome (LFL), which are a familial clustering of early onset tumors, such as adrenal cortical carcinomas, brain tumors, breast cancers, and sarcomas [232,233]. 

Similar to somatic mutations, germline substitutions are mostly missense mutations (75.2%) [230,231]. Although somatic mutations primarily affect the DBD (residues 101–300) (93.1%) and the OD (residues 326–356) (1.6%) [152], the distribution of germline mutations among p53 domains is somewhat different: their frequency in the DBD is relatively lower (72.7%) and the frequency in the OD is much higher (19.6%) than those of the somatic mutations [152]. The OD mutation found in LFS and LFL families are Arg333Cys, Arg337Cys/His/Pro, Arg342Pro, and Ala347Asp [232,233,234,235,236,237,238,239,240,241].

Modern literature dedicated to p53 is vast. As of 18 September 2016, there were 82,267 p53-related papers in PubMed, of which 8920 papers were specifically dedicated to p53 mutants. There were 591 reviews on p53 mutants. Obviously, space restraints do not allow for providing an in-depth analysis of the functional and pathological consequences of p53 mutations that are known to affect the conformational stability of DBD, modulate the oligomerization potential of p53, influence its ability to interact with DNA and partner proteins, and have profound effects on various biological functions of this protein. Since non-focused general analysis of this subject would undoubtedly be taken as superficial, this section of the review will be limited to a very general overview of how mutations can be related to the proteoform concept. 

Analysis of data reported in the UMD TP53 mutation database (http://p53.fr/) revealed that the total number of p53 mutations is 36,249, of which 29,035, 3030, 476, and 3707 are missense, nonsense, splice, and frame shift mutations, respectively. Figure 10C shows that mutations can affect almost all residues of this protein. In fact, 96% of p53 residues are known to be mutated. All exceptions are located within the intrinsically disordered N- and C-terminal tails of p53 and include Glu_3_, Pro_4_, Phe_19_, Ser_20_, Leu_22_, Trp_23_, Val_97_, Ala_335_, Lys_372_, Gly_374_, Ser_378_, Met_384_, Lys_386_, Glu_388_, Pro_390_, and Asp_391_. The typical logic behind establishing correlations between mutations causing functional alteration and cancer pathogenesis is based on finding “hotspots”; i.e., specific sites of a protein that are most commonly affected by tumor mutations, since missense mutations of functionally important residues would abolish or compromise functions and therefore these mutations should be frequently observed in tumors. In this model, residues whose mutations are not common or not found at all in tumors are typically considered as not essential. An alternative viewpoint on this phenomenon is that residues not affected by disease-causing mutations represent “do not touch me” sites, mutations of which could be lethal. 

The pink bars in Figure 10C represent the distribution of disease-causing mutations within the p53 sequence. This part of the plot is shown in semi-logarithmic scale, since the number of disease mutations found for a giving residue of p53 ranges from 0 to >1000 for some of the hotspots (e.g., the DBD-located Arg_175_, Arg_273_, and Arg_248_ have 1092, 1425, and 1544 such mutations, respectively). This plot has a hill-like shape, reflecting that more mutations can be found in the central, more ordered part of the protein than at its more disordered ends. To illustrate the heterogeneity of missense mutations, we looked at the sequence distribution of the number of different missense mutations affecting a given p53 residue. The value of this parameter ranges from 1 to 13, and the corresponding plot also has a hill-like shape (see the blue bars in Figure 10C). Finally, the red bars show the heterogeneity of missense germline mutations associated with LFS and LFL syndromes and annotated in UniProt (http://www.uniprot.org/uniprot/ P04637#pathology_and_biotech).

Analysis of the data shown in Figure 10 delivers several important messages:

(1) Almost all residues of p53 are subject to disease-related mutations;
(2) The ordered central part of this protein has more mutations than its disordered tails;
(3) The heterogeneity of missense mutations is also higher for the central region of p53;
(4) Both order-promoting and disorder-promoting residues can be efficiently mutated. In fact, numbers of mutations found for the order-promoting Cys_176_ and Tyr_220_ (343 and 324, respectively) are not too different from the corresponding values reported for the disorder-promoting residues Arg_213_ and Pro_278_ (327 and 268, respectively). Also, the hottest of p53 hotspots (i.e., residues that have >500 mutations each) are the disorder-promoting residues Arg_248_, Arg_273_, Arg_175_, Gly_245_, Arg_282_, and Arg_249_, with mutations reported 1544, 1425, 1092, 718, 616, and 573 times, respectively;
(5) The abundance and diversity of mutations for a given residue depend mostly on its position within the p53 sequence and not so much on its physico-chemical nature;
(6) Mutations tend to “like” disorder-based binding regions, but are preferentially excluded from the majority of the PTM sites.

These observations suggest that, in comparison with other factors able to create p53 proteoforms, mutations generate the largest number of proteoforms by heterogeneously altering the majority of p53 residues. This defines the remarkable functional and structural variability of mutation-generated proteoforms, which can not only annul the tumor-suppressive functions of p53, but can also generate neomorphic p53 proteoforms by bestowing novel activities on the mutated protein [242].

### 7.4. The More the Merrier: Complex p53 Proteoforms Created by Mutations, PTMs, and Alternative Splicing

Obviously, all factors described in the previous sections (alternative splicing, PTMs, and mutations) can be combined to further enhance the structure–functional heterogeneity of p53 and generate even more complex proteoforms of this protein. In relation to PTMs, mutations can have three possible outcomes: (a) they can eliminate PTMs by replacing modifiable residues by non-modifiable ones; (b) they can change the “personality” of a residue by making it susceptible to the non-native type of PTM; or (c) they can synergize with PTMs to change the protein functionality. 

One of the first studies of PTMs in mutated p35 revealed that the phosphorylation of Ser15 and Ser392 in p53 mutants associated with human tumors was altered compared to the wild-type p53 [243]. It was pointed out that several residues subjected to PTMs in normal p53 are affected by cancer-causing mutations [216]. For example, the Arg_337_ residue, which can be dimethylated, is considered a hotspot for *TP53* germline mutations that present as adrenocortical tumors [244,245]. Similarly, mutations of the phosphorylatable residues Thr155 and Ser215 located in the DBD are found 99 and 110 times in tumors, respectively [231]. Even in the absence of stress, the C-terminally located Ser392 is hyperphosphorylated in several tumor-derived cell lines containing the Arg248Trp or Arg273His hotspot mutations [243,246,247].

Although the majority of p53 residues subjected to PTM are infrequently mutated in human tumors, and although none of the PTM sites found in the p53 DBD represents a hotspot for tumor mutations, the codons for Lys_132_, Thr_155_, Ser_215_, Glu_258_, Asp_259_, and Cys_277_ were shown to have at least 90 cancer-associated mutations each [216]. These observations indicate that the posttranslationally modifiable residues are important but not vital for the tumor suppressor and genome guardian roles of p53. This conclusion is supported by the finding that most mutations affecting PTM-bearing residues only partially compromise p53 transactivation [216], and by the results of the in vitro mutagenesis of each of the p53 residues that can be subjected to PTMs, where no significant reduction of p53 transactivation activities was found in a yeast-based assay [248].

Unfortunately, not much is known about the regulatory roles of PTMs in modulating the functionality of p53 mutants. Furthermore, it was pointed out that the majority of existing data addressing this issue are not very reliable, since they are obtained using in vitro models with non-physiological expression levels of p53 or transformed cell lines with altered signaling pathways that influence p53 activities [216]. The limited data from animal models suggest that some PTMs could be important for malignant functions of mutant p53 [216,249]. It is expected that phosphorylation of mutant forms of p53 might affect the pattern of other PTMs in the protein or induce conformational changes that would affect biological activities of mutated p53, such as its interactions with other proteins and, potentially, responses to anticancer drugs [216]. For example, the RAS-mediated phosphorylation of the Arg280Lys mutant of p53 at Ser6 and Ser9 promotes interaction of the mutated p53 with Smad, and the formation of this complex results in the inhibition of the antimetastasis activities of p63 [250]. Mutations of the phosphorylatable Ser15 and Ser46 residues in the N-terminal region of p53 are related to the variability of the radiosensitivity of lung cancer cells [251]. The JNK-mediated phosphorylation of the N-terminus or the PLK2-driven phosphorylation of C-terminus leads to noticeable enhancement of the oncogenic gain of function of the mutated p53 [252,253].

Of the 11 acetylatable lysine residues, only three (Lys_120_, Lys_164_, and Lys_305_) are often found mutated in cancer, with two of those being located within the DBD [216]. Several p53 mutants (e.g., Arg248Trp and Arg273His) were shown to be characterized by the enhanced acetylation of Lys_320_, Lys_373_, or Lys_382_, even in the absence of stress [246,247]. Analysis of several cancer cell lines harboring mutated forms of p53, such as ovarian TOV cells possessing the Arg175His p53 mutation, pancreatic cancer PANC1 cells with the Arg280Thr mutation, breast carcinoma MDA-MB-231 cells with the Arg280Lys p53 mutation, T47D cells with Lys194Phe, and BT20 cells with Lys132Gln, revealed that the glucose restriction induces acetylation of C-terminally located lysines in the mutated but not in the wild-type p53, suggesting that regulation of the autophagic process in response to starvation is lost in tumor cells [254]. Another interesting observation, that acetylation of non-functional p53 mutant forms Arg175His and Gly245Ala was able to partially restore their DNA-binding and growth suppression activities, suggests that PTMs not only can regulate the activity of mutated p53 proteoforms but can also control their affinities for DNA [255]. 

In addition to phosphorylation and acetylation, cancer-related mutations in p53 were shown to have profound effects on the efficiency of its ubiquitination, resulting in the hyperstability of p53 that might be responsible for the oncogenic activities of mutated proteoforms of this protein [216]. For example, it was established that tumor-derived endogenous cell lines harboring different p53 mutations were characterized by a complete lack of ubiquitination and showed a dramatic increase (10- to 20-fold above cancer cell lines harboring wild-type p53) in the constitutive stabilization of p53 [256].

As was pointed out, p53 isoforms, which are generated via alternative initiation of translation, usage of alternative promoters, and alternative splicing, all share DBD as a common part and contain different N-terminal TADs and CTDs. Although regardless of *TP53* mutation status in cancer cells, abnormal expression of p53 isoforms is known to play an active role in the formation and progression of cancer [165], questions about how often p53 isoforms are affected by cancer-related mutations and how various p53 activities can be modulated by mutating alternative isoforms remain open. However, knowing the detrimental effects of mutations and abnormal expression of p53 isoforms, one can only imagine what damage can be caused by proteoforms generated as a result of the combined action of these two factors. Another level of complexity will be added considering the possibility of generating induced proteoforms by combining alternative splicing, mutations, and PTMs. Of course, on top of all that one should keep in mind the exceptional conformational heterogeneity of p53 caused by its intrinsic disorder and structural flexibility, which will further enhance the complexity of the realm of p53 proteoforms.

## 8. Conclusions

In a recent review article discussing the roles of various p53 mutations in modulating the structure and function of this protein, it was pointed out that “While many groups choose to use the generic term “mutant p53” to designate any tumor-derived p53 mutant, it is important to recognize that not all p53 mutants are equal” [242]. This conclusion was based on the observation that some mutations are able to generate neomorphic p53 proteins with novel functions and it led to the statement that “mutant p53” represents a case of a “one name, many proteins” model [242]. 

This conclusion is obviously relying on the “one-gene–one-protein–one-function” paradigm, where manifestation of a new function should serve as a reflection of the appearance of a new protein. However, in my view, since any protein (including p53) exists in a cell as a dynamic ensemble of different proteoforms, and since the spectrum of such proteoforms is very complex due to the presence of multiple mechanisms that can act on a protein individually or in various combinations, p53 should be considered an important and compelling illustration of the protein structure–function continuum concept. In fact, p53 exists as an ensemble of multiple different proteoforms that are generated by several means, ranging from the intrinsically disordered nature of its N- and C-termini generating basic or conformational proteoforms to interaction with binding partners shifting the equilibrium in the p53 conformational ensemble, and thereby generating a set of functioning proteoforms; to alternative splicing, alternative promoter usage, and alternative initiation of translation producing at least 12 alternative proteoforms; to different PTMs that can affect more than 60 residues of this protein, generating a realm of differently modified proteoforms containing single or multiple PTMs of a similar or different nature; and to cancer-associated mutations that can affect 96% of p53 residues, providing them with new identities (with ~87% of p53 residues able to gain multiple personalities bestowed on them by different missense mutations). 

**Figure 11:**
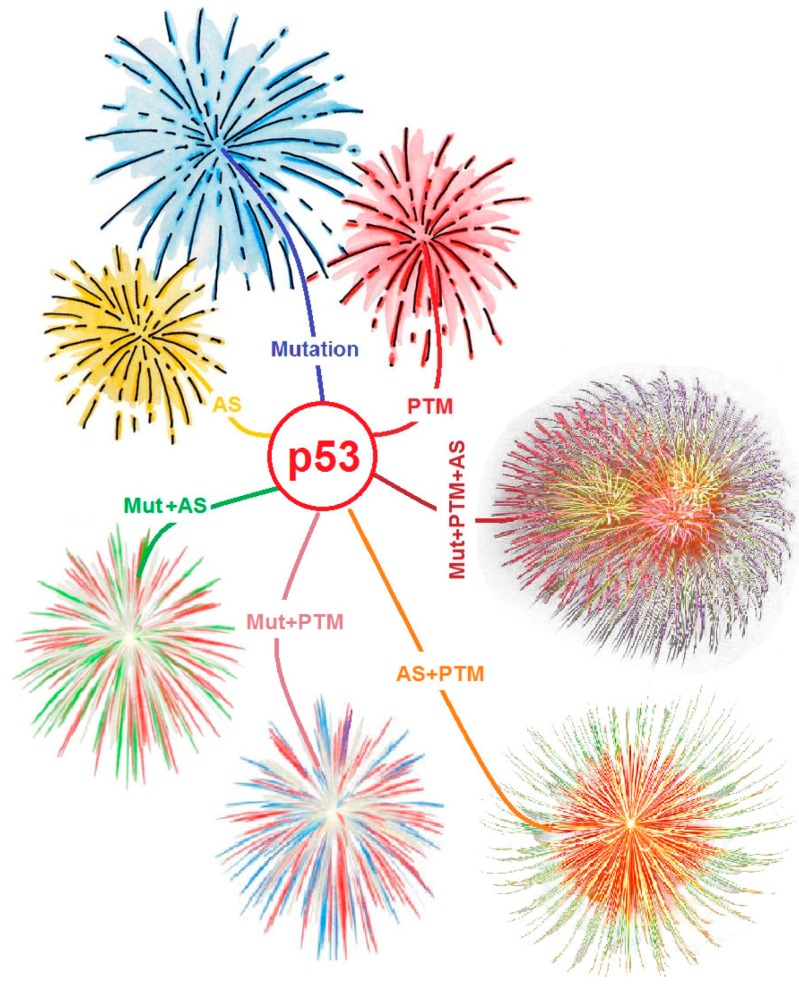
Fireworks model of the p53 proteoforms. Here, proteoforms generated by alternative splicing (AS), or mutations, or posttranslational modifications (PTM) are shown by yellow, blue and red colors, respectively. More complex proteoforms generated by mutations and alternative splicing (Mut + AS), mutations and PTMs (Mut + PTM), AS and PTM (AS + PTM), or mutations, PTMs and AS (Mut + PTM +AS) are shown as fireworks of mixed colors.

The remarkable complexity of the p53 system is illustrated by Figure 11, which represents a small subset of inducible p53 proteoforms in the form of fireworks with different traces reflecting modifications of the p53 sequence produced by various proteoform-inducing mechanisms (mutation, PTM, alternative splicing) and with differently colored “explosions” representing the conformational ensembles originating from the dynamic nature of the p53 molecule. Here, p53 is shown to have a mutation-induced conformational proteoform (blue explosion), a PTM-induced form (red explosion), an alternative splicing (AS)-induced form (yellow explosion), three conformational proteoforms of p53 sequences generated by the combined action of a single mutation and a single PTM (red–blue explosion), a single mutation and a single AS event (green–red explosion), and a single AS event and a single PTM (red-yellow explosion), and a conformational proteoform generated by the combined action a single mutation, a single PTM, and a single AS event (red–blue–yellow explosion). In reality, the situation is much more complex, since the p53 sequence can be affected by numerous PTMs, mutations, and AS events that can act individually or in various combinations. Therefore, continuing the firework analogy, the complete picture of the p53 structure–function space originating from the existence of multiple proteoforms generated by different mechanisms can be depicted as a night sky that is brightly illuminated by the fireworks finale, when multiple fireballs are shot simultaneously.

It is important to emphasize here that although p53 was used in this article to illustrate the essence of the protein structure–function continuum concept, this protein by no means represents an exception. Of course, finding examples of various aspects related to this concept was simplified by the profusion of available p53-related studies and publications. However, in my view, similar (maybe a bit less detailed) essays can be easily drafted for several other pathology-associated proteins, such as cancer-related PTEN, BRCA1, androgen receptor, DMP1, Mdm2, proteins from the Wnt pathway, and HER2, or neurodegeneration-related α-synuclein, FUS, TDP43, huntingtin, and tau protein, to name a few. The reason for listing these proteins here is simple—they popped out from the crowd, being described as “troublemakers” that trigger some abnormal processes. In fact, for them (as well as many other proteins), it was shown that an aberration in any part of the cellular machinery generating proteoforms (which are the basis of the existence of the protein structure–function continuum) might have some disastrous consequences [194]. For example, it is now recognized that deregulation of splicing events (via mutations or misexpression of some splicing factors) is a crucial trigger and initiator of carcinogenesis and tumor progression [257]. Obviously, this list can be further extended to include almost any protein.

## Supplementary Materials

Supplementary materials can be found at www.mdpi.com/1422-0067/17/11/1874/s1.
