# Supplementary material for: p53 Proteoforms and Intrinsic Disorder: An Illustration of the Protein Structure–Function Continuum Concept
Source: Int J Mol Sci. 2016 Nov 10;17(11):1874. doi: 10.3390/ijms17111874 (PMC5133874; doi:10.3390/ijms17111874)
Supplement: Supplementary file 1 [file ijms-17-01874-s001.pdf]

Supplementary Materials: p53 Proteoforms and Intrinsic Disorder: An Illustration of the Protein Structure-Function Continuum Concept

Vladimir N. Uversky

Table S1. 141 X-ray and NMR structures of various human p53 fragments.

Entry	Method	Resolution (Å)	Chain	Positions	Partners
**2K8F ***	**NMR**	**-**	**B**	**1–39**	**p300 TAZ2 domain (1723–1812; Q09472)**
**5HOU**	**NMR**	**-**	**A**	**1–61**	**Fusion with TAZ1 domain of CBP**
**5HPD**	**NMR**	**-**	**A**	**2–61**	**Fusion with TAZ2 domain of CBP**
1YCQ	X-ray	2.30	B	13–29	Mdm2 from Xenopus laevis (12–118; P56273)
**2L14**	**NMR**	**-**	**B**	**13–61**	**CBP nuclear coactivator binding domain (2061–2117, P45481)**
**2LY4**	**NMR**	**-**	**B**	**1–93 (14–60)**	**HMGB1 (1–83; P09429). Note: structure is available for the 14–60 fragment of p53 only**
**1YCR**	**X-ray**	**2.60**	**B**	**15–29**	**Mdm2 from Homo sapiens (17–125; Q00987)**
**2MWY**	**NMR**	**-**	**B**	**15–29**	**Mdm4 from Homo sapiens (23–111; O15151)**
2Z5S	X-ray	2.30	P/Q/R	15–29	Mdm4 from Danio rerio (14–139; Q7ZUW7)
2Z5T	X-ray	2.30	P/Q/R	15–29	Mdm4 from Danio rerio (14–139; Q7ZUW7)
3DAB	X-ray	1.90	B/D/F/H	15–29	Mdm4 from Homo sapiens (23–111; O15151)
4HFZ	X-ray	2.69	B/D	15–29	Mdm2 from Homo sapiens (17–125; Q00987)
**3DAC**	**X-ray**	**1.80**	**B/P**	**17–37**	**Mdm4 from Danio rerio (15–129; Q7ZUW7)**
**2GS0**	**NMR**	**-**	**B**	**20–73 (45–58)**	**RNA polymerase II transcription factor B subunit 1 (residues 1–115; P32776). Note: structure is available for the 45–58 fragment of p53 only**
**2B3G**	**X-ray**	**1.60**	**B**	**33–60**	**Replication protein A 70 kDa DNA-binding subunit (residues 1–120; P04637)**
2MZD	NMR	-	B	35–59	p300 TAZ2 domain (1723–1812; Q09472)
5HP0	NMR	-	A	37–61	Fusion with TAZ1 domain of CBP
**2RUK**	**NMR**	**-**	**A**	**41–62**	**TFIIH p62 PH domain (1–108; P32780)**
2XWR	X-ray	1.68	A/B	89–293	Self; p53 DNA binding domain (89–293; P04637)
4HJE	X-ray	1.91	A/B/C/D	92–291	DNA
3KMD	X-ray	2.15	A/B/C/D	92–291	DNA

Table S1. *Cont.*

Entry	Method	Resolution (Å)	Chain	Positions	Partners
2H1L	X-ray	3.16	M/N/O/P/Q/R/S/T/U/V/W/X	92–292	Helicase Domain of Large T antigen from Macaca mulatta polyomavirus 1 (260–627; P03070)
4QO1	X-ray	1.92	B	92–312	Nb139 nanobody against the DNA-binding domain of p53
1YCS	X-ray	2.20	A	94–292	SH3 domain of 53BP2 (327–519; Q13625)
4MZI	X-ray	1.25	A	94–292	Alone; Mutation: S121F, V122G, C135V, C141V, W146Y, C182S, V203A, R209P, C229Y, H233Y, Y234F, N235K, Y236F, T253V, N268D
2PCX	X-ray	1.54	A	94–292	Alone; R282Q mutant
4XR8	X-ray	2.25	C/D	94–292	Maltose-binding periplasmic protein, ubiquitin ligase E6AP (27–107, P0AEX9) and E6 (8–86, P03126)
2AC0	X-ray	1.80	A/B/C/D	94–293	DNA
2ADY	X-ray	2.50	A/B	94–293	DNA
2AHI	X-ray	1.85	A/B/C/D	94–293	DNA
2ATA	X-ray	2.20	A/B/C/D	94–293	DNA
2YBG	X-ray	1.90	A/B/C/D	94–293	p53 with acetylated Lysine 120
3D05	X-ray	1.70	A	94–293	Alone; R249S mutant
3D06	X-ray	1.20	A	94–293	Alone; R249S mutant
3D07	X-ray	2.20	A/B	94–293	Alone; R249S mutant
3D08	X-ray	1.40	A	94–293	Alone; R249S/H168R mutant
3D09	X-ray	1.90	A	94–293	Alone; R249S/H168R/T123A mutant
3D0A	X-ray	1.80	A/B/C/D	94–293	DNA; R249S/H168R mutant
3IGK	X-ray	1.70	A	94–293	DNA; R249S/H168R mutant
3IGL	X-ray	1.80	A	94–293	DNA; wild type (wt)
3KZ8	X-ray	1.91	A/B	94–293	DNA
4IBQ	X-ray	1.80	A/B/C/D	94–293	Alone; R273C mutant
4IBS	X-ray	1.78	A/B/C/D	94–293	Alone; R273H mutant
4IBY	X-ray	1.45	A/B	94–293	Alone; R273H/S240R mutant
4IBT	X-ray	1.70	A/B/C/D	94–293	Alone; R273H/T284R mutant
4IBZ	X-ray	1.92	A/B/C/D	94–293	Alone; R273C/T284R mutant
4IBU	X-ray	1.70	A/B/C/D	94–293	DNA; R273C/T284R mutant
4IBV	X-ray	2.10	A	94–293	DNA; R273C/S240R mutant

Table S1. *Cont.*

Entry	Method	Resolution (Å)	Chain	Positions	Partners
4IBW	X-ray	1.79	A	94–293	DNA; R273H/T284R mutant
2J1Y	X-ray	1.69	A/B/C/D	94–293	Alone; M133L/V203A/N239Y/G245S/N268D mutant
4IJT	X-ray	1.78	A	94–293	Alone; R273H mutant
**2FEJ**	**NMR**	**-**	**A**	**94–297**	**Alone**
4KVP	X-ray	1.50	A/B/C/D	94–312	Alone; V157F mutant
4LO9	X-ray	2.50	A/B/C/D	94–312	Alone; N235K mutant
4LOE	X-ray	1.85	A/B/C/D	94–312	Alone; N239Y mutant
4LOF	X-ray	2.00	A	94–312	Alone; V157F/N235K/N239Y mutant
1TSR	X-ray	2.20	A/B/C	94–312	DNA; wt
1TUP	X-ray	2.20	A/B/C	94–312	DNA; wt
1UOL	X-ray	1.90	A/B	94–312	Alone; M133L/V203A/N239Y/N268D mutant
2BIM	X-ray	1.98	A/B	94–312	Alone; M133L/V203A/N239Y/N268D/R273H mutant
2BIN	X-ray	1.90	A	94–312	Alone; M133L/H168R/V203A/N239Y/N268D mutant
2BIO	X-ray	1.90	A	94–312	Alone; M133L/V203A/N239Y/R249S/N268D mutant
2BIP	X-ray	1.80	A	94–312	Alone; M133L/H168R/V203A/N239Y/R249S/N268D mutant
2BIQ	X-ray	1.80	A	94–312	Alone; T123A/M133L/H168R/V203A/N239Y/R249S/N268D mutant
2J1W	X-ray	1.80	A/B	94–312	Alone; M133L/V143A/V203A/N239Y/N268D mutant
2J1X	X-ray	1.65	A/B	94–312	Alone; M133L/V203A/Y220C/N239Y/N268D mutant
2J1Z	X-ray	1.80	A/B	94–312	Alone; M133L/V203A/N239Y/N268D/F270L mutant
2J20	X-ray	1.80	A/B	94–312	Alone; M133L/V203A/N239Y/N268D/R273C mutant
2J21	X-ray	1.60	A/B	94–312	Alone; M133L/V203A/N239Y/N268D/R282W mutant
2WGX	X-ray	1.75	A/B	94–312	Alone; M133L/V203A/Y236F/N239Y/T253I/N268D mutant
2OCJ	X-ray	2.05	A/B/C/D	94–312	Alone; wt
2VUK	X-ray	1.50	A/B	94–312	Stabilizing small molecule PhiKan083; Y220C mutant
2X0U	X-ray	1.60	A/B	94–312	2-amino substituted benzothiazole scaffold; Y220C mutant
2X0V	X-ray	1.80	A/B	94–312	4-(trifluoromethyl)benzene-1,2-diamine; Y220C mutant
2X0W	X-ray	2.10	A/B	94–312	5,6-dimethoxy-2-methylbenzothiazole; Y220C mutant
3ZME	X-ray	1.35	A/B	94–312	Stabilizing small molecule PhiKan7242; Y220C mutant
4AGL	X-ray	1.70	A/B	94–312	Stabilizing small molecule PhiKan784; Y220C mutant

Table S1. *Cont.*

Entry	Method	Resolution (Å)	Chain	Positions	Partners
4AGM	X-ray	1.52	A/B	94–312	Stabilizing small molecule PhiKan5086; Y220C mutant
4AGN	X-ray	1.60	A/B	94–312	Stabilizing small molecule PhiKan5116; Y220C mutant
4AGO	X-ray	1.45	A/B	94–312	Stabilizing small molecule PhiKan5174; Y220C mutant
4AGP	X-ray	1.50	A/B	94–312	Stabilizing small molecule PhiKan5176; Y220C mutant
4AGQ	X-ray	1.42	A/B	94–312	Stabilizing small molecule PhiKan5196; Y220C mutant
5A7B	X-ray	1.40	A/B	94–312	Stabilizing small molecule PhiKan5211; Y220C mutant
5ABA	X-ray	1.62	A/B	94–312	Stabilizing small molecule PhiKan5149; Y220C mutant
5AOK	X-ray	1.35	A/B	94–312	Stabilizing small molecule PhiKan7099; Y220C mutant
5AOM	X-ray	1.74	A/B	94–312	Stabilizing small molecule PhiKan883; Y220C mutant
5G4M	X-ray	1.38	A/B	94–312	Monofluorinated derivative of Phikan083; Y220C mutant
5G4N	X-ray	1.35	A/B	94–312	Difluorinated derivative of Phikan083; Y220C mutant
5G4O	X-ray	1.48	A/B	94–312	Trifluorinated derivative of Phikan083; Y220C mutant
5AB9	X-ray	1.36	A/B	94–312	7-ethyl-3-(piperidin-4-yl)-1H-indole; Y220C mutant
5AOI	X-ray	1.78	A/B	94–312	Indole-based small molecule; Y220C mutant
5AOJ	X-ray	1.47	A/B	94–312	2-hydroxy-3,5-diiodo-4-(1H-pyrrol-1-yl)benzoic acid; Y220C mutant
5AOL	X-ray	1.50	A/B	94–312	3-bromo-5-(trifluoromethyl)benzene-1,2-diamine; Y220C mutant
3Q01	X-ray	2.10	A/B	94–356	Homodimer; C135V, C141V, W146Y, C182S, V203A, R209P, C229Y, H233Y, Y234F, N235K, Y236F, T253V, N268D, P322T, L323M, M340Q, L344R mutant
3Q05	X-ray	2.40	A/B/C/D	94–356	DNA; C135V, C141V, W146Y, C182S, V203A, R209P, C229Y, H233Y, Y234F, N235K, Y236F, T253V, N268D, P322T, L323M, M340Q, L344R, G356T mutant
3TS8	X-ray	2.80	A/B/C/D	94–356	Natural CDKN1A(p21) p53-response element (DNA); C135V, C141V, W146Y, C182S, V203A, R209P, C229Y, H233Y, Y234F, N235K, Y236F, T253V, N268D, P322T, L323M, M340Q, L344R, G356T mutant
**4MZR**	**X-ray**	**2.90**	**A/B/C/D**	**94–388**	**DNA; S121F, V122G, C135V, C141V, W146Y, C182S, V203A, R209P, C229Y, H233Y, Y234F, N235K, Y236F, T253V, N268D, ΔK292, ΔG293, ΔE294, ΔP295, ΔH296, ΔH297, ΔE298, ΔL299, ΔP300, ΔP301, ΔG302, ΔS303, ΔT304, ΔK305, ΔR306, ΔA307, ΔL308, ΔP309, ΔN310, ΔN311, ΔS313, ΔS314, ΔS315, ΔP316, ΔQ317, ΔP318, ΔK319, ΔK320, P322T, L323M, M340Q, L344R, G356T, K357E mutant**

Table S1. *Cont.*

Entry	Method	Resolution (Å)	Chain	Positions	Partners
1KZY	X-ray	2.50	A/B	95–289	p53-binding protein 1 (1714–1972; Q12888)
1GZH	X-ray	2.60	A/C	95–292	p53-binding protein 1 (1724–1972; Q12888)
5ECG	X-ray	3.00	A/B	95–312	p53-binding protein 1 (1724–1972; Q12888) and SpQEY peptide of the histone H2A variant H2AX
2MEJ	NMR	-	B	96–312	Bcl-2-like protein 1 (1–209, Q07817)
3Q06	X-ray	3.20	A/B/C/D	96–354	DNA
**2YDR**	**X-ray**	**2.75**	**P**	**144–154**	**O-GlcNAcase NAGJ (31–618, Q0TR53)**
**4RP6**	**X-ray**	**1.70**	**Z**	**252–258**	**Amyloid-like fibril**
4RP7	X-ray	1.58	Z	253–258	Amyloid-like fibril
1OLG	NMR	-	A/B/C/D	319–360	Homotetramer
**1OLH**	**NMR**	**-**	**A/B/C/D**	**319–360**	**Homotetramer**
1SAE	NMR	-	A/B/C/D	319–360	Homotetramer
1SAF	NMR	-	A/B/C/D	319–360	Homotetramer
1SAK	NMR	-	A/B/C/D	319–360	Homotetramer
1SAL	NMR	-	A/B/C/D	319–360	Homotetramer
3SAK	NMR	-	A/B/C/D	319–360	Homotetramer
1HS5	NMR	-	A/B	324–357	Designed homodimer, Met340Gln/Leu344Arg mutant
1A1U	NMR	-	A/C	324–358	Designed homodimer, Met340Lys/Phe341Ile/ Leu344Tyr mutant
1PET	NMR	-	A/B/C/D	325–355	Homotetramer
1PES	NMR	-	A/B/C/D	325–355	Homotetramer
1C26	X-ray	1.70	A	325–356	Homotetramer
2J0Z	NMR	-	A/B/C/D	326–356	Homotetramer, T329F/Q331K mutant
2J10	NMR	-	A/B/C/D	326–356	Homotetramer, T329F/Q331K mutant
2J11	NMR	-	A/B/C/D	326–356	Homotetramer, Y327S/T329G/Q331G mutant
1AIE	X-ray	1.50	A	326–356	Homotetramer
**2FOO**	**X-ray**	**2.20**	**B**	**358–363**	**N-terminal domain of HAUSP/USP7 (51–205; Q93009)**
2F1X	X-ray	2.30	A/B	359–368	N-terminal domain of HAUSP/USP7 (49–209; Q93009)
2FOJ	X-ray	1.60	B	361–367	N-terminal domain of HAUSP/USP7 (51–209; Q93009)

Table S1. *Cont.*

Entry	Method	Resolution (Å)	Chain	Positions	Partners
**2MWO**	**NMR**	**-**	**B**	**363–377**	**Tumor suppressor p53-binding protein 1 (Tudor-like region residues 1484–1603, Q12888)**
**3TG5**	**X-ray**	**2.30**	**B**	**365–375**	***N*-lysine methyltransferase SMYD2 (Q9NRG4) and SAH (*S*-adenosyl-L-homocysteine)**
**1JSP**	**NMR**	**-**	**A**	**367–386**	**CBP Bromodomain (1081–1197, Q92793)**
**1DT7**	**NMR**	**-**	**X/Y**	**367–388**	**Calcium-bound S100ββ (P04631)**
**1XQH**	**X-ray**	**1.75**	**B/F**	**369–377**	**Methyltransferase SET9 (107–366, Q8WTS6) and SAH**
1MA3	X-ray	2.00	B	372–389	Transcriptional regulatory protein Sir2 from Archaeoglobus fulgidus (O30124)
**1YC5**	**X-ray**	**1.40**	**B**	**372–389**	**NAD-dependent deacetylase Sir2 from Thermotoga maritima (Q9WYW0)**
2H2D	X-ray	1.70	B	372–389	NAD-dependent deacetylase Sir2 from Thermotoga maritima (Q9WYW0)
2H2F	X-ray	2.20	B	372–389	NAD-dependent deacetylase Sir2 from Homo sapience (P04637)
2H4F	X-ray	2.00	D	372–389	NAD-dependent deacetylase Sir2 from Thermotoga maritima (Q9WYW0)
2H4H	X-ray	1.99	B	372–389	NAD-dependent deacetylase Sir2 from Thermotoga maritima (Q9WYW0), H116Y mutant
2H4J	X-ray	2.10	D	372–389	NAD-dependent deacetylase Sir2 from Thermotoga maritima (Q9WYW0)
2H59	X-ray	1.90	D/E	372–389	NAD-dependent deacetylase Sir2 from Thermotoga maritima (Q9WYW0), H116A mutant
3PDH	X-ray	1.80	D	372–389	NAD-dependent deacetylase Sir2 from Thermotoga maritima (Q9WYW0)
1H26	X-ray	2.24	E	376–386	Cell division protein kinase 2 (CDK2, P24941) and Cyclin A2 (173–432, P20248)
**2MWP**	**NMR**	**-**	**B**	**376–387**	**Tumor suppressor p53-binding protein 1 (Tudor-like region residues 1484–1603, Q12888)**
**4BV2**	**X-ray**	**3.30**	**E/H**	**376–388**	**NAD-dependent deacetylase Sir2 from Thermotoga maritima (Q9WYW0)**
3OQ5	X-ray	2.50	D/E	377–386	3-MBT domain from human Lethal(3)malignant brain tumor-like protein, L3MBTL1 (191–530, Q9Y468). Note: entire sequence of the p53 fragment is missing in this structure
**4X34**	**X-ray**	**1.80**	**C/D**	**377–386**	**Tumor suppressor p53-binding protein 1 (Tudor-like region residues 1484–1603, Q12888)**
**4ZZJ**	**X-ray**	**2.74**	**B**	**379–383**	**NAD-dependent protein deacetylase sirtuin-1 (183–503, Q96EB6)**
**4BUZ**	**X-ray**	**1.90**	**P**	**379–386**	**NAD-dependent deacetylase Sir2 from Thermotoga maritima (Q9WYW0)**
**3LW1**	**X-ray**	**1.28**	**P**	**385–393**	**14-3-3 protein sigma (P31947)**

* Entries indicated by the bold font represent structures discussed in the text.
